# Hetero-Diels–Alder and CuAAC Click Reactions for Fluorine-18 Labeling of Peptides: Automation and Comparative Study of the Two Methods

**DOI:** 10.3390/molecules29133198

**Published:** 2024-07-05

**Authors:** Timothé Maujean, Sridévi M. Ramanoudjame, Stéphanie Riché, Clothilde Le Guen, Frédéric Boisson, Sylviane Muller, Dominique Bonnet, Mihaela Gulea, Patrice Marchand

**Affiliations:** 1 Université de Strasbourg, CNRS, Laboratoire d’Innovation Thérapeutique, LIT UMR 7200, F-67000 Strasbourg, France; 2 Inovarion, F-75005 Paris, France; 3 Université de Strasbourg, CNRS, Institut Pluridisciplinaire Hubert Curien, IPHC UMR 7178, F-67000 Strasbourg, France; 4 Université de Strasbourg, CNRS, Biotechnologie et Signalisation Cellulaire UMR 7242, F-67000 Strasbourg, France

**Keywords:** fluorine-18, peptide, cycloaddition, hetero-Diels–Alder, CuAAC, labeling, radiochemistry, automated synthesis

## Abstract

Radiolabeled peptides are valuable tools for diagnosis or therapies; they are often radiofluorinated using an indirect approach based on an F-18 prosthetic group. Herein, we are reporting our results on the F-18 radiolabeling of three peptides using two different methods based on click reactions. The first one used the well-known CuAAC reaction, and the second one is based on our recently reported hetero-Diels–Alder (HDA) using a dithioesters (thia-Diels–Alder) reaction. Both methods have been automated, and the ^18^F-peptides were obtained in similar yields and synthesis time (37–39% decay corrected yields by both methods in 120–140 min). However, to obtain similar yields, the CuAAC needs a large amount of copper along with many additives, while the HDA is a catalyst and metal-free reaction necessitating only an appropriate ratio of water/ethanol. The HDA can therefore be considered as a minimalist method offering easy access to fluorine-18 labeled peptides and making it a valuable additional tool for the indirect and site-specific labeling of peptides or biomolecules.

## 1. Introduction

Peptides are biomolecules exhibiting high binding affinities to specific receptors, which explains their use for therapeutic application (alone or combined to a β- or α emitting radioisotope) but also as diagnostic tools when tagged with fluorescent, radioactive, magnetic or enzyme probes.

During the development of therapeutic peptides, radiolabeling represents an attractive method to assess pharmacokinetics and pharmacodynamics data associated with whole-body biodistribution quantification using non-invasive imaging. When optimal properties are reached, labeled peptides can also be used as diagnostic tools in abnormal biological processes or to follow disease progression/regression. For all those reasons, there has been a growing interest for years toward the development of peptide radiolabeling strategies using the whole set of available isotopes [1,2,3,4,5,6,7].

Among the isotopes available, fluorine-18, used for positron emission tomography (PET) imaging, is one of the most widely used due to its favorable physical properties (branching ratio, energy of the β^+^) and its ease of production in a cyclotron. Moreover, its half-life (t_1/2_) of 109.7 min is in perfect adequacy with the kinetics of peptide biodistribution, justifying its extensive use in this field [8,9,10,11,12,13,14].

Peptides are sensitive molecules, and mild labeling conditions are mandatory. Some innovative radiolabeling methods via direct fluorination have been recently reported [15,16,17,18,19,20,21], but despite their interest, the indirect strategy based on the use of a ^18^F-prosthetic group remains the method of choice for site-specific radiolabeling [22,23,24,25,26,27,28,29,30]. Ideally, labeling reactions should be performed with good kinetics, high conversion, and total chemoselectivity to limit by-products formation. All those prerequisites match with the definition of click reactions explaining their wide use in this field [31,32,33,34]. In the click chemistry toolbox, cycloadditions such as the well-known CuAAC (copper-catalyzed azide-alkyne cycloaddition) [35,36,37,38,39], metal-free reactions like the SPAAC (strain-promoted azide-alkyne cycloaddition) [40,41,42,43,44,45], IEDDA (inverse electron demand Diels–Alder) [46,47,48,49,50,51,52], or cycloadditions of mesoionic compounds [53,54,55] represent interesting reactions for peptide radiolabeling. When working with peptides, the CuAAC strategy remains the most often used despite copper and/or ascorbate mediated oxidation reactions on amino acid side chains related to the formation of reactive oxygen species [56,57,58]. The catalyst-free methods mentioned above also exhibit several drawbacks including difficult access to one or both partners, which is particularly related to the introduction of appropriate functionalities on peptides. In this context, we recently reported a catalyst-free hetero-Diels–Alder (HDA) reaction of dithioesters (thia-Diels–Alder) for the ^18^F-labeling of peptides. As a proof of concept, we applied it to the low molecular weight pseudo-peptide PSMA (KuE: lysine-urea-glutamate an ureido peptide of 320 g·mol^−1^) [59].

Herein, we describe a comparative study of indirect ^18^F-labeling of three different peptides (apelin **3**, apelin-C_8_F_17_ **4** and P140 **5**) by two methods, which are both fully automatized: one using the CuAAC method on peptide-azides and the other by the HDA approach on peptide-dithioesters (Scheme 1 and Supplementary Materials Figures S1–S12, chemical structures of peptides).

## 2. Results

### 2.1. Chemistry

#### 2.1.1. Non-Peptidic Cycloaddition Partners

- for CuAAC

**Scheme 2:**
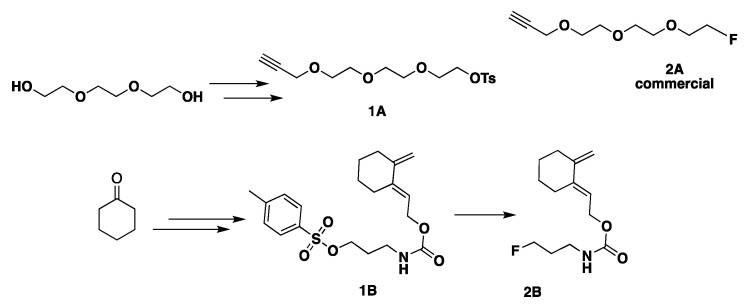
Synthesis of tosylate precursors **1A**, **1B** and non-radioactive fluorinated **2A** and **2B**.

The propargylic tosylate **1A** (2-(2-(2-(prop-2-yn-1-yloxy)ethoxy)ethoxy)ethyl 4-methylbenzenesulfonate, Scheme 2) was prepared on gram scale in two steps following a described method [60]. ^1^H NMR and ^13^C NMR analyses were in good agreement with the published data. The non-radioactive fluorinated **2A** was purchased from Advanced Biochemical Compounds (ABX, Radeberg, Germany, CAS [1003005-81-9]) and was used as received for the click reaction to obtain the reference non-radioactive peptides.

- for HDA reaction

Exocyclic tosylated diene **1B** (Scheme 2) was synthesized according to the previously described method in six steps starting from cyclohexanone [59]. Starting from **1B**, by substitution of the tosylate using Bu_4_NF in refluxing acetonitrile, the fluorinated diene **2B** was obtained after purification in 75% yield. The synthesis was carried out on gram scale.

Both tosylates **1A** and **1B** were stable over at least 18 months when stored at −20 °C.

#### 2.1.2. Synthesis of Peptides and Modified Peptides

- Synthesis of modified azido-peptides **3A**–**5A** for CuAAC

Peptides were obtained by automated solid-phase peptide synthesis (SPPS) using standard Fmoc/*tert*-butyl solid-phase chemistry on a preloaded Wang resin (0.67 mmol·g^−1^ resin, 0.1 mmol scale) and using DMF as solvent, diisopropylcarbodiimide (DIC) and Oxyma as coupling agent for each amino acid insertion. Fmoc groups were removed using a 20% *v*/*v* solution of piperidine in DMF. Washing steps were performed using DMF.

*N*-terminus functionalization was performed using the dried resin previously synthesized by automated SPPS. For **3A** and **4A**, a protected azido lysine was used to introduce the N_3_ reactive moiety. Peptide **4A** was further elongated on the lysine (after Fmoc deprotection using a 20% (*v*/*v*) solution of piperidine in DMF) by coupling 4,4,5,5,6,6,7,7,8,8,9,9,10,10,11,11-heptadecafluoroundecanoic acid (using HATU and DIEA in DMF). Peptide **5A** was obtained by coupling on the unprotected *N*-terminus a 5-azidopentanoic acid (activated with HATU and DIEA). Peptides were cleaved from the resin under reducing acidic conditions with TFA/H_2_O/phenol/thioanisole/EDT. After filtration and precipitation, the peptides were purified by preparative reversed-phase HPLC. and collected fractions were lyophilized. Peptides **3A**–**5A** were obtained with average yields ranging from 40 to 45% and characterized by LC/MS (Supplementary Materials protocols pages 6–7).

- Synthesis of modified dithioester-peptides **3B**–**5B** for HDA reactions

Peptides were synthesized by automated SPPS as described above. After deprotection of the *N*-terminus, supported peptides on the support were coupled to 4-(bromomethyl)benzoic acid using DIC/DMAP in CH_2_Cl_2_. After washing, diethoxy-phosphonodithioformate salt was introduced for the S-alkylation step. Cleavage of the resin was performed using only TFA/TIS (97/3 *v*/*v*) to avoid degradation of the dithioester by the nucleophilic 1,4-dimercapto-2,3-butanediol (DTT). After evaporation, the peptides were precipitated in diethyl ether, filtered and then dissolved for HPLC purification. Peptides **3B**–**5B** were obtained with an average yield of 30% as pink solids after freeze-drying (Supplementary Materials pages 8–10).

- Synthesis of reference peptides **6**–**8**

Non-radioactive fluorinated reference peptides (**6A**–**8A**) for CuAAC were synthesized by mixing pre-activated CuSO_4_ (pre-mixed with sodium ascorbate and TBTA (tris(benzyltriazolylmethyl)amine) as ligand in water) with the azido-peptides (**3A**–**5A**) and the commercial fluoro-propargylic derivative **2A** (in a mixture of DMF/water at 37 °C). After HPLC purification and freeze-drying of the collected fractions, peptides were obtained with an average yield of 10%. The low yields obtained were due to the non-optimization of the CuAAC reaction conditions and the difficult HPLC separation between products (**6A**–**8A**) and azido-peptides (**3A**–**5A**) when reactions were not complete (Supplementary Materials pages 7–8).

For the HDA reactions, the reference peptides **6B**–**8B** were prepared by reacting dithioester-peptides (**3B**–**5B**) with **2B** in water/isopropanol (70/30 *v*/*v*) at 60 °C. After completion of the reaction, products were isolated by C18 semi-preparative HPLC, and the fractions were freeze-dried to give the reference peptides with an average isolated yield of 36% (Supplementary Materials pages 10–11).

### 2.2. Radiochemistry

#### 2.2.1. Manual Synthesis

**Scheme 3:**
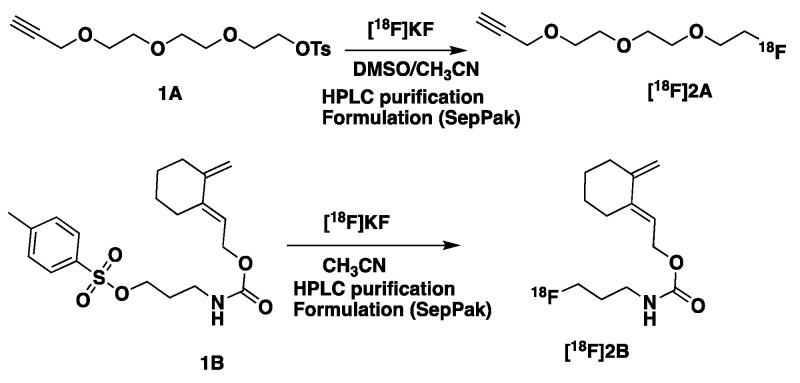
Radiosynthesis of prosthetic groups **[^18^F]2A** for CuAAC and **[^18^F]2B** for HDA.

- Synthesis of [^18^F]**2A** and [^18^F]**2B** (Scheme 3, see also Supplementary Materials pages 12–13)

Manual syntheses with a low level of radioactivity (10–30 MBq) were performed to optimize the reaction conditions for [^18^F]**2A** and [^18^F]**2B**. Starting from 3 mg of tosylate **1A**, [^18^F]-**2A** was obtained with radiochemical conversion (RCC) of more than 90% in CH_3_CN/DMSO (450/350 μL) using [^18^F]KF-K_2.2.2_ (0.7 mg of K_2_CO_3_ and 9 mg of K_2.2.2_) at 115 °C during 10 min (Supplementary Materials Figure S13).

For [^18^F]**2B**, 5 mg of tosylate **1B** was used in CH_3_CN (1 mL) with 0.6–0.65 mg of K_2_CO_3_ and 12 mg of K_2.2.2_. After 10 min at 110 °C, a radiochemical conversion (RCC) of 90% up to 100% (Supplementary Materials Figure S14) was obtained [60].

- Optimization of the CuAAC reaction using peptide **3A**

**Scheme 4:**
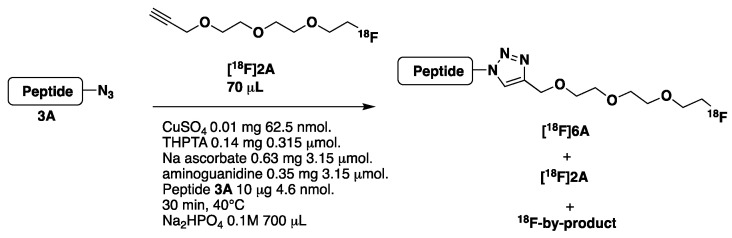
Reaction conditions using THPTA as ligand for the synthesis of **[^18^F]6A**.

The first set of reactions was performed manually and monitored by HPLC using the azido-peptide **3A**. [^18^F]-**2A** was produced on an automated synthetizer and used after HPLC purification and formulation in ethanol (Supplementary Materials Figure S15). Table 1 summarizes the results obtained with or without THPTA ((tris(3-hydroxypropyltriazolylmethyl)amine)) as the ligand in the presence or absence of peptide **3A**. The reaction conditions are depicted in Scheme 4.

Entry 1 and 2 (Table 1) proved not only the inefficiency of the CuAAC when using THPTA as the ligand but also the almost quantitative formation of an unknown radioactive by-product. This by-product (Rt = 11.9 min) was observed in the presence or the absence of the azido-peptide. It was not formed when the prosthetic group **[^18^F]2A** was placed with the reagents (CuSO_4_, aminoguanidine, sodium ascorbate) in the absence of THPTA (Supplementary Materials Figure S16 and protocol page 14).

**Scheme 5:**
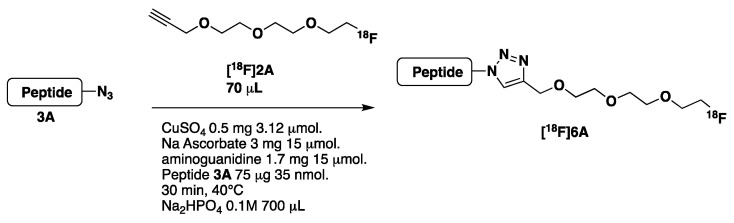
Optimized conditions for manual radiosynthesis of **[^18^F]6A**.

The conditions had then to be optimized without a ligand (mainly by increasing the copper salts content). A radiochemical conversion of 98.8% was obtained when heating at 40 °C during 30 min a solution containing 0.5 mg of CuSO_4_ (3.12 μmol), sodium ascorbate for copper(II) reduction (3 mg, 15 μmol), aminoguanidine used to trap reactive species of reduced ascorbate (1.7 mg, 15 μmol) and 75 μg (35 nmol) of peptide **3A** in 700 μL of Na_2_HPO_4_ (0.1 M) mixed with 70 μL of [^18^F]**2A** in ethanol (Scheme 5, see also Supplementary Materials page 15 and Figure S17).

**Scheme 6:**
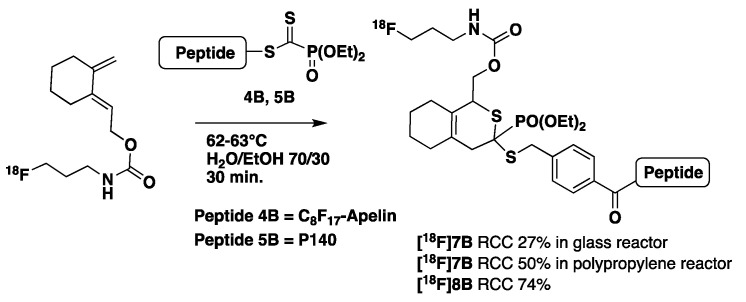
Optimized conditions for manual radiosynthesis of **[^18^F]7B** and **[^18^F]8B**.

- Optimization of the HDA reaction (Scheme 6)

The cycloaddition was assessed on peptides **4B** and **5B** using conditions previously optimized for PSMA. A batch of **[^18^F]2B** (formulated in ethanol) was produced on an automated synthetizer (1 GBq·mL^−1^, Supplementary Materials Figure S18). For the manual reaction, 4.5 μg (1.5 nmol) of **5B** (in water) was mixed with 5 μL of **[^18^F]2B** in ethanol. The solution was adjusted to 70 μL to reach a water/ethanol ratio of 70/30 (*v*/*v*). After heating at 62–63 °C for 30 min, a radiochemical conversion of 74% (**[^18^F]-8B**) was obtained (radio HPLC, 17% of remaining **[^18^F]2B** and 9% of impurities, Supplementary Materials Figure S19).

The reaction was carried out in the same conditions with peptide **4B** in a glass tube, and after 30 min, a radiochemical conversion of 27% was obtained (with 7% impurities and 65% of remaining **[^18^F]2B**). The tendency of apelin-C_8_F_17_ to stick on glass walls is well known in our laboratory, and therefore, the reaction was tested again under exactly the same conditions but in a 2 mL polypropylene tube. A conversion of 50% was obtained in 30 min at 62–63 °C (remaining **[^18^F]2B** 40%, impurities 10%), and 63% radiochemical conversion was obtained after 45 min of reaction (Supplementary Materials pages 16–17 and Figure S20).

#### 2.2.2. Automated Synthesis

The CuAAC and the HDA methods were both automatized on a SynChrom EVO III module (Raytest, Straubenhardt, Germany) using the reaction conditions determined by manual optimization. The automated processes include the preparation of the prosthetic group (**[^18^F]2A** or **[^18^F]2B**) up to the formulation of purified [^18^F]-peptides.

Table 2 gathers the conditions and the results of the automated processes. For **[^18^F]2A**, the purification was performed by C18 HPLC, and the collected fraction (up to 13 mL) was further dissolved in water before being trapped on two HLB Oasis (Waters) solid-phase extraction cartridges (Sep-Pak) for re-concentration. The use of a single HLB Sep-Pak or two classical C18 Sep-Paks proved to be insufficient for an efficient trapping (loss of radioactivity was observed during the process when using a single Sep-Pak). **[^18^F]2A** was then released from the cartridges using 1.6 mL of acetone; the evaporation of acetone was mandatory for the next step and also permits to concentrate the prosthetic group within a few minutes. Attempts to re-concentrate ethanolic solutions of **[^18^F]2A** led to an important degradation of the prosthetic group. For the second step, the CuAAC, we increased the amount of CuSO_4_·5H_2_O from 0.5 mg in manual synthesis to 0.8 mg for automated reactions, and sodium gentisate (2 mg) was added to avoid side reactions (radiolysis and oxidation). The temperature was increased to 55 °C in the automated version of the CuAAC.

Reactions were carried out with 0.3–0.5 mg of peptides (**3A**–**5A**, 0.1 μmol) in a minimal volume (1.2 mL) of water/DMF (90/10 *v*/*v*) or phosphate buffer 0.05 M (pH 7.5). For peptide **4A** (C_8_F_17_-Apelin-N_3_), we observed a low reactivity in phosphate solution, but the reactivity was restored (37% isolated yield of **[^18^F]7A**) in water/DMF. All peptides (**[^18^F]6A**–**[^18^F]8A**) were purified and reformulated by C18 Sep-Pak. Crude solutions were treated with EDTA (20 mg in water) for dilution and removal of copper, passed on a C18 cartridge and then washed using Et_2_O. Even if using diethyl ether on a C18 cartridge is an uncommon procedure, it works efficiently to remove the unreacted prosthetic group and some of the organic additives used in the CuAAC. A second wash was added (H_2_O/EtOH 80/20 *v*/*v*) before the elution of the peptides **[^18^F]6A**–**[^18^F]8A** (with the remaining **3A**–**5A**) by 1.1 mL of ethanol containing 1% of HCl 0.2 M. On an irregular basis, we observed a progressive degradation (radio HPLC) of the labeled peptides with the formation of a by-product (up to 20% within less than 2 h). To circumvent this issue, the formulation was modified by the addition of sodium gentisate (80 mg) and methionine (15 mg) to the final solution (total volume 11 mL, ready for injection), and products were found to be stable up to 6 h (Supplementary Materials page 25, Figure S28) after the end of the synthesis with high radiochemical purities (above 95%). Decay corrected yields of 30–47% were obtained (Supplementary Materials pages 18–19, Scheme S1 and Figure S21).

Automation of the HDA method was straightforward due to its simplicity. The prosthetic group **[^18^F]2B** was purified by HPLC and reformulated by C18 Sep-Pak in 1.45 mL of ethanol for the next step. All attempts to re-concentrate the solution, using diethyl ether, acetone, ethanol or a mixture of solvents, failed, and low to very low conversions were observed (most probably due to radiolysis of the prosthetic group during re-concentration).

Reactions were carried out with an average of 3 mg of peptides (**3B**–**5B**, 0.8 μmol) in water/ethanol. Even if diluted up to 5.2 mL to respect the water/ethanol ratio of 70/30, the reaction proceeded as expected, and the conversion of more than 75% was observed after 45 min of reaction at 62 °C. The labeled peptides **[^18^F]6AB**–**[^18^F]8B** were purified by HPLC to remove the excess of peptide-dithioesters **3B**–**5B**. The collected fraction was reformulated with a C18 Sep-Pak, and the products were eluted from the cartridge with a solution of ethanol (1.15 mL) and HCl 0.2M (30 μL). The three peptides were obtained with a radiochemical purity superior to 95% and a decay corrected yield of 30–40% (Supplementary Materials pages 20–21, Scheme S2 and Figure S22), and they were found to be stable in solution (10–11 mL, ready to inject) for a minimum of 6 h after the end of synthesis (Supplementary Materials page 29, Figure S35).

## 3. Discussion

We have developed two fully automated methods for the ^18^F-labeling of peptides. They involve two types of cycloadditions belonging to the click toolbox: the first one based on the widely used CuAAC reaction and the second one using a hetero-Diels–Alder (HDA) reaction recently reported by us [59]. Both methods represent indirect approaches using fluorine-18 labeled prosthetic groups and have been applied on three different peptides to allow a general but also head-to-head comparison. Three peptides were selected for this study. The first one was apelin-17 (**3**, also known as K17F), a truncated form of apelin, which is a natural agonist ligand of the apelin receptor (APJ receptor that belongs to the large family of G-protein coupled receptors, GPCRs) with potential applications for the treatment of cardiovascular diseases, glucose regulation and the development of blood vessels [61]. The second peptide was C_8_F_17_-apelin (**4**), a modified apelin-17 derivative exhibiting high affinity for APJ receptors and a much higher in vitro and in vivo stability than apelin-17 thanks to the stabilizing C_8_F_17_ chain, which was grafted on the *N*-terminus [62]. The last selected peptide was P140 (**5**), a 21-mer peptide derived from the U1-70K splicesosomal protein, which contains a mandatory phosphorylated serine at position 140. P140 exhibits a large set of beneficial effects in various autoimmune and inflammatory diseases [63]. It was extensively studied and proved to regulate and correct abnormal autophagic activities [64]. Some derivatives of those peptides (P140 and apelin-13) were previously tagged with fluorescent groups, and more recently, a 68-Ga labeling of apelin-13 was reported [64,65,66].

The automation of ^18^F-radiolabeling procedures is nowadays a common method when dealing with small molecules but is rarely used for the radiolabeling of peptides or more complex molecules [67]. Most of the reported peptide radiolabeling is based on a two-step sequence with one step or even both performed manually. In addition to an increased exposure to radiation, this strategy will inherently be restricted to the production of small activities. Many recent automates can accommodate complex syntheses and provide large number of radiotracers, even peptides, for high-throughput biological evaluation and potential translation of the labeled molecules for human imaging.

The CuAAC has been applied to small molecular weight molecules up to nanoparticles producing a wide diversity of ^18^F-labeled molecules. Interestingly, the partners can be reversed and when considering peptides, amino acids modified with a propargyl or an azide group are available, and they can be inserted during the solid-supported production of peptides. The same is true for the second partner of the click reaction, and many derivatives can be synthesized or are commercially available [68,69]. We decided to use azido-peptides and to react them with a propargylic triethylene glycol derivative. Peptides were synthesized on solid support and further modified by the introduction of an azido-lysine on the *N*-terminus for **3A**, **4A** and with a pentanoic azide for **5A**. The propargylic triethylene glycol (**[^18^F]2A**) was chosen for its in vivo stability, its solubility in water and organic solvents and also for its higher boiling point than shorter glycol or propargylic alkanes. **[^18^F]2A** was previously involved in “hot” CuAAC reactions with small molecular weight azides or even an azido-RGD peptide [70,71,72]. In all cases, a large amount of copper salt was used (1.5–2.5 mg) with 1–2 mg of azide to reach moderate to good yields (30–69% decay corrected yields).

The CuAAC method when applied to biomolecules and peptides is prone to generate by-products arising from oxidation (mainly on methionine, cysteine, tyrosine and histidine) or side reactions between lysine, arginine or guanidine and the oxidized form of sodium ascorbate [56,57,58]. The use of copper ligands can drastically limit those side reactions and decreases the amount of necessary copper salts in this reaction. However, in most cases, a final HPLC purification would be necessary to remove the ligands. In our study, we chose to explore the CuAAC using THPTA, a water-soluble copper ligand, that could be removed by a simple solid phase extraction (Sep-Pak) purification. Initial reactions (carried out manually), using THPTA, gave disappointing results even though similar conditions have been previously described [35]. We observed an almost quantitative formation of an unknown ^18^F by-product resulting from the presence of THPTA and CuSO_4_/ascorbate on the prosthetic group **[^18^F]2A** (see Table 1). This by-product could be attributed to the Glaser homo-coupling of the propargylic groups even if reactions were carried out with capped vials to avoid di-oxygen interactions [39]. We therefore turned to more classical conditions (without ligands) adapted from a protocol previously described for the ^18^F-labeling of propargylic-peptides using a [^18^F]triethyleneglycol azide [37]. The use of copper sulfate in the absence of any ligand forced us not only to increase considerably the total amount of copper (up to 0.8 mg of copper salt for only 0.3–0.5 mg of peptide) but also the amount of other reagents (Na ascorbate for copper reduction and amino-guanidine to trap reactive species of reduced ascorbate). Advantageously, the absence of ligand and the use of a small amount of azido-peptides (**3A**–**5A**) allowed us to perform the purification and the formulation of **6A**–**8A** simply on a C18 solid phase extraction cartridge (C18 Sep-Pak) with optimized conditions to remove unreacted **[^18^F]2A**. The automated process (Supplementary Materials Scheme S1 and Figure S21) includes the preparation of **[^18^F]2A** and its purification. As the prosthetic group was stable enough and non-volatile, it was possible after HPLC and reformulation in acetone (after trapping it on Sep-Pak) to concentrate it almost to dryness (presence of residual water from last wash of the SepPak). The reaction could then be performed in a minimal volume of solvent (1.2 mL) increasing therefore the concentration of reagents and thus the kinetic of the reaction. Reactions were carried out in phosphate solutions except for peptide **4A**, where a low reactivity was observed due to the self-assembly of the C_8_F_17_-apelin in phosphate buffer [73]. The solvent was thus changed to a mixture of water/DMF (90/10) which restored the reactivity of the peptide, giving **[^18^F]7A** with an isolated yield of 37%. Starting from 22 +/− 2 GBq (*n* = 16) of fluorine-18, an average of 8–10 GBq of **[^18^F]2A** were isolated in 54 +/− 2 min. The molar activity was not determined due to the very low absorbance of **[^18^F]2A** but was estimated to be in the range of 75–100 GBq/μmol (values usually obtained on the same automate, within our laboratory, for other single-step fluorination reactions). Therefore, each CuAAC experiment was carried out with an amount of 0.08–0.13 μmoles of **[^18^F]2A** approximately along with a slight excess of each peptide **3A**–**5A** (0.3–0.5 mg, 0.1–0.15 μmol). As the peptides were used only in slight excess, the purification could be performed by Sep-Pak. Even if peptides **3A**–**5A** could not be separated by such a technique from the ^18^F-peptides (**[^18^F]6A**–**[^18^F]8A**), only a low amount of azido-peptides was present in the final product. We observed, but not on a regular basis, the formation of by-products during the reaction and also after the final purification. Thus, we added a small amount of sodium gentisate (2 mg, as suggested by others) [37] to the reaction mixture, and we modified the formulation step by the addition of sodium gentisate and methionine as anti-radiolytic excipients and scavengers of oxidative species. In such conditions, the automated process produced a few GBq (mean activity collected 4.1 GBq +/− 1.2 GBq, *n* = 15) of the ^18^F-peptides **[^18^F]6A**, **[^18^F]7A** and **[^18^F]8A** with radiochemical purities exceeding 95% and an average yield (decay corrected) of 39% within a synthesis time of 120 min (Supplementary Materials, chromatograms of purified ^18^F-peptides Figures S23–S27).

Automation of the HDA process was much more straightforward due to the simplicity of the reaction and our previous experience when labeling a PSMA derivative [59]. Peptides (**3B**–**5B**) were modified on solid support to introduce the dithioester group and were found to be highly stable when stored freeze-dried as trifluoroacetate salts. We first attempted, as for the CuAAC to re-concentrate the prosthetic group **[^18^F]2B** after HPLC purification and trapping on C18 Sep-Pak. Whatever the solvents used, only low radiochemical conversions were observed for the subsequent click reaction step. The elution from the Sep-Pak cartridges was therefore optimized not to exceed a total volume of 1.45 mL of alcohol; this allowed us to perform the HDA reaction in a maximum volume of 5.2–5.3 mL (corresponding to the volume of the HPLC injection loop) while respecting the optimum ratio of water/ethanol (70/30 *v*/*v*). Starting from 26.6+/− 1.3 GBq of fluorine-18, the ^18^F-labeled diene **[^18^F]2B** was obtained in 52 min with a molar activity (Am) up to 150 GBq/μmol and a total activity of 9–10 GBq after HPLC purification and C18 Sep-Pak reformulation in ethanol. For the C_8_F_17_-apelin derivative (**4B**), the setup was modified to accommodate a polypropylene reactor (homemade from a 10 mL polypropylene falcon tube) to avoid adsorption of the peptide on glassware. As 3 mg (0.8 μmol) of peptide-dithioesters **3B**–**5B** had to be used for efficient reaction, we added an HPLC purification of the final product.

The automated process was performed in an average time of 149 min despite the two HPLC purifications and provided the ^18^F-peptides **[^18^F]6B**, **[^18^F]7B** and **[^18^F]8B** with radiopurities above 95% (Supplementary Materials, chromatograms of purified [^18^F]-peptides Figures S29–S34), a decay corrected yield of 37.6% (*n* = 13) and an average molar activity (Am) of 53.5 GBq/μmol (*n* = 10, min. 14, max. 79 GBq/μmol).

## 4. Materials and Methods

Reagents were obtained from commercial sources and used without any further purification unless otherwise stated. Low-resolution mass spectra (LRMS) and high-resolution mass spectra (HRMS) were obtained on an Agilent Technologie 6520 Accurare-Mass Q.Tof LC/MS (Agilent Technologies, Les Ulis, France) apparatus equipped with a Zorbax SB C18 column (1.8 μm, 2.1 × 50 mm, Agilent Technologies, Les Ulis, France) using electrospray ionization (ESI) and a time-of-flight analyzer (TOF).

All reagents for radiochemistry were used without further purification. K_2_CO_3_ 99.99%, Kryptofix K_2.2.2_ (4,7,13,16,21,24-hexaoxa-1,10-diazabicyclo-[8.8.8]-hexacosane), anhydrous acetonitrile 99.8%, ethanol (absolute, HPLC grade), hydrochloric acid, trifluoroacetic acid (HPLC grade) and sodium acetate (analytical grade) were purchased from Merck (Darmstadt, Germany). Accell plus QMA carbonate light cartridges were obtained from Waters (Milford, MA, USA, 130 mg sorbent, Part No 186004051) and used as received, C18 Sep-Pak (tC18 environmental WAT 036800 and C18 WAT 020515) was purchased from Waters and pre-conditioned with 5 mL of MeCN or 5 mL of ethanol followed by 10 mL of pure H_2_O before use. OASIS HLB Sep-Pak was obtained from Waters and conditioned with 5 mL of MeCN followed by 10 mL of pure H_2_O. [^18^O]H_2_O ([^18^O] 98%) for [^18^F]fluoride production was purchased from Nukem isotopes GmbH-Germany (Alzenau, Germany). Pure H_2_O (18.2 MΩ) was produced with a Purelab option Q purification system (Veolia Water Technologies, Saint-Maurice, France). Sodium chloride 0.9% sterile solution was purchased from B BRAUN medical (Melsungen, Germany).

[^18^F]-fluoride production was performed with an ACSI^®^ 24 MeV cyclotron, Advanced Cyclotron System Inc., Richmond, BC, Canada by the proton irradiation of a 1 mL volume niobium target at an energy of 16.5 MeV and an intensity of 35 μA. After 5 min of cooling, the radioactivity was transferred to the hot cell under helium pressure; then, the transfer lines and the target were rinsed twice (2 × 1 mL) with pure water. The total transferred activity was measured (well counter in the hot cell) and further transferred to the Raytest module reception vial under helium pressure. Residual activity in the intermediate vial after transfer was counted to determine the activity used in each radiosynthesis (a typical activity of 23–24 GBq for a 11 μA irradiation was transferred in the hot cell).

Analytical HPLC was performed on an HPLC Dionex^®^ U3000, Life Technologies SAS, Villebon-sur-yvette, France equipped with a DAD detector and a radioactivity detector using C18 Kinetex (Phenomenex, Torrance, CA, USA, 5 μm EVO 4.6 × 150 mm) or Syncronis (250 × 4.6 mm 5 µm, Thermo Fisher Scientific, Macquarie Park, Australia) analytical columns with gradient of MeCN/H_2_O + 0.1% TFA.

Full experimental procedures can be found in the Supplementary Materials File.

## 5. Conclusions

We have developed the automated ^18^F-radiolabeling of three peptides of therapeutic interest by using two types of cycloadditions, the HDA reaction of dithioester-peptides and the well-known CuAAC reaction of azido-peptides. The obtained results allowed us to critically compare the two methods. In terms of yields and synthesis time, both reactions compared equally. However, to reach similar results, the CuAAC reaction necessitated a large loading of copper salts (30 equivalents/azido-peptide) along with sodium ascorbate, aminoguanidine and sodium gentisate but only a small amount of peptide (0.3–0.5 mg, 0.1–0.15 μmol). By comparison, the HDA is a minimalistic reaction needing only a correct ratio of solvents, no catalyst or additive, but requiring a larger amount of peptides (3 mg, 0.8 μmol) to proceed with similar kinetics. Moreover, some by-products were observed during or after the CuAAC reaction, while no side products were ever observed in the HDA reaction. It is, however, worth noting that the CuAAC reaction offers more flexibility, as both partners can be inverted (peptide-alkyne and fluoro-azide), while it is not yet the case for the HDA reaction.

The comparison of the two methods allows us to conclude that the HDA of dithioesters, even if not perfect due to the amount of peptide engaged in the reaction, represents a simple and effective approach for the ^18^F-labeling of peptides and an interesting alternative method to access radiolabeled biomolecules under soft conditions.

## Figures and Tables

**Scheme 1:**
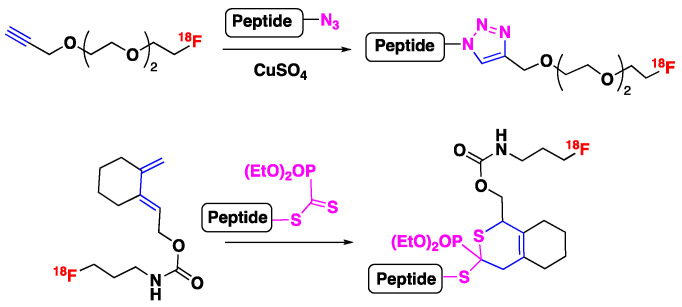
General scheme of the two cycloadditions for ^18^F-labeling of peptides. Top: CuAAC on azido-peptide. Bottom: HDA reaction using a peptide-dithioester.

**Table 1:** Reaction conditions and radio-HPLC quantifications of the product [^18^F]**6A**, starting material [^18^F]**2A**, and main by-product with and without THPTA as ligand.

Entry	Reagents	[^18^F]6A ^3^ Rt = 13.3 min	[^18^F]2A ^3^ Rt = 15.7 min	^18^F-by-Product ^3^ Rt =11.9 min
1	CuSO_4_, THPTA, Na Ascorbate, Aminoguanidine, peptide **3A**	9.7%	0.15%	90.2%
2 ^1^	CuSO_4_, THPTA, Na Ascorbate, Aminoguanidine ^1^	0%	1.2%	98.8%
3 ^2^	CuSO_4_, Na Ascorbate, Aminoguanidine ^2^	0%	91.8%	0%

^1^ The reaction was performed without peptide **3A**. ^2^ Reaction without peptide **3A** and without ligand (THPTA). ^3^ Percentages determined by C18 HPLC on radioactive channel.

**Table 2:** Reaction conditions and isolated yields of ^18^F-peptides in CuAAC and HDA reactions using fully automated process.

Method	Step 1	Step 2	Mean Isolated Yield DC ^1^ (NDC) ^2^	Time (min)
CuAAC	**1A** 3 mg CH_3_CN/DMSO, 10 min 115 °C HPLC + C18 Sep-Pak and evaporation	Peptide **3A**, **4A**, **5A**, 0.3–0.5 mg/**[^18^F]-2A** CuSO_4_ (0.8 mg), Na Ascorbate (6.3 mg) aminoguanidine (3.6 mg), sodium gentisate (2 mg) H_2_O/DMF (90/10 V =1.2 mL) or PB ^3^ 0.05 M (1.2 mL) 55 °C, 30 min C18 Sep-Pak purification	**[^18^F]6A** 47% (22%) *n* = 7 **[^18^F]7A** 37% (17.5%) *n* = 3 **[^18^F]8A** 30% (13.5%) *n* = 5 mean DC 39.3% *n* = 15	120
HDA	**1B** 5 mg CH_3_CN, 10 min 110 °C HPLC + C18 Sep-Pak	Peptide **3B**, **4B** ^4^, **5B**, 2.5–3.5 mg/**[^18^F]-2B** H_2_O/EtOH (70/30, V = 5.2 mL) HPLC + C18 Sep-Pak	**[^18^F]6B** 30% (12.7%) *n* = 3 **[^18^F]7B** 38.7% (15%) *n* = 4 **[^18^F]8B** 40% (14.8%) *n* = 6 mean DC 37.6% *n* = 13	145

^1^ DC decay corrected yield of purified product from start of synthesis. ^2^ NDC non-decay corrected yield of purified product. ^3^ PB: phosphate buffer 0.05 M pH 7.5 (only for peptide **3A**, see Supplementary Materials p12 for details on phosphate buffer composition). ^4^ HDA reaction performed in a polypropylene reactor.

## Data Availability

Data are contained within the article and Supplementary Materials.

## Supplementary Materials

The following supporting information can be downloaded at https://www.mdpi.com/article/10.3390/molecules29133198/s1; Figures S1–S12: Chemical structures of azido-peptides (**3A**–**5A**), peptide-dithioesters (**3B**–**5B**) and ^18^F-peptides (**[^18^F]6A**–**8A**, **[^18^F]6B**–**8B**); Detailed protocols for peptide syntheses (**3A**–**5A**, **3B**–**5B**, non-radioactive references **6A**–**8A** and **6B**–**8B** and mass analyses), Radiochemistry protocols and HPLC analyses for manual and automated syntheses (Figures S13–S22 and Schemes S1–S2); HPLC analyzes of purified peptides (**[^18^F]6A**–**8A** and **[^18^F]6B**–**8B** and stability over 6 h (Figures S23–S35).
