# Supplementary material for: Hetero-Diels–Alder and CuAAC Click Reactions for Fluorine-18 Labeling of Peptides: Automation and Comparative Study of the Two Methods
Source: Molecules. 2024 Jul 5;29(13):3198. doi: 10.3390/molecules29133198 (PMC11243578; doi:10.3390/molecules29133198)
Supplement: Supplementary file 1 [file molecules-29-03198-s001.zip › molecules-3057870-supplementary.pdf]

SUPPLEMENTARY MATERIALS

Hetero-Diels–Alder and CuAAC click reactions for fluorine-18 labeling of peptides: Automation and comparative study of the two methods

Timothé Maujean [1], Sridévi M. Ramanoudjame [1], Stéphanie Riché [1], Clothilde Le Guen [1,2], Frédéric Boisson [3], Sylviane Muller [4], Dominique Bonnet [1], Mihaela Gulea [1] and Patrice Marchand [3,*]

[1] Université de Strasbourg, CNRS, Laboratoire d'Innovation Thérapeutique, LIT UMR 7200, F-67000 Strasbourg, France
[2] Inovarion, F-75005 Paris, France
[3] Université de Strasbourg, CNRS, Institu Pluridisciplinaire Hubert Curien, IPHC UMR 7178, F-67000 Strasbourg, France
[4] Université de Strasbourg, CNRS, Biotechnologie et signalisation cellulaire UMR 7242, F-67000 Strasbourg, France
* Correspondence: patrice.marchand@iphc.cnrs.fr

Table of content

Chemistry *2*
Chemical structures of peptides: 2
Generalities (chemistry) 5
Synthesis of labeling precursors 1A and 1B and non radioactive 2A and 2B 6
Peptide-N_3 synthesis (3A-4A-5A) for CuAAC 6
Non-radioactive reference Peptides 7
Peptide-dithioesters synthesis (3B-4B-5B) for HDA 8
Non-radioactive reference Peptides 10
RADIOCHEMISTRY *11*
Generalities (radiochemistry) 11
Manual synthesis of [^{18}F]2A 12
Manual synthesis of [^{18}F]2B 13
Optimization of the CuAAC reactions using peptide 3A 13
Reaction with THPTA (Main text, table 1 entry 1-3): 13
Optimized conditions of the CuAAC reaction (manual) with peptide 3A. 15
Optimized conditions of the HDA reaction with peptides 4B and 5B 15
HDA reaction of 5B in glass reactor 16
HDA reaction of 4B in glass reactor 16
HDA reaction of 4B in polypropylene reactor 16
Automated Synthesis *17*
Generalities 17
Fully automated CuAAC on peptides 3A, 4A, 5A. 17
Fully automated HDA on peptides 3B, 4B, 5B. 19
HPLC analyses of purified peptides and co-injection with non radioactive references *22*
HPLC analyses of peptides [^{18}F]6A-8A (CuAAC) 22
Stability of peptides [^{18}F]6A-8A in ready to inject solution 6 hours after the end of synthesis: 25
HPLC analyses of peptides [^{18}F]6B-8B (HDA) 26
Stability of peptides [^{18}F]6B-8B in ready to inject solution 6 hours after the end of synthesis: 29

Chemistry

Chemical structures of peptides:

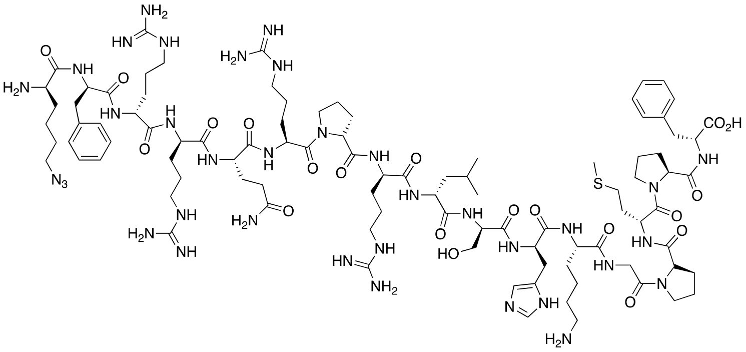

Figure S1: Chemical structure of peptide **3A** [H-K(N_3)FRRQRPRLSHKGPMPF-OH]

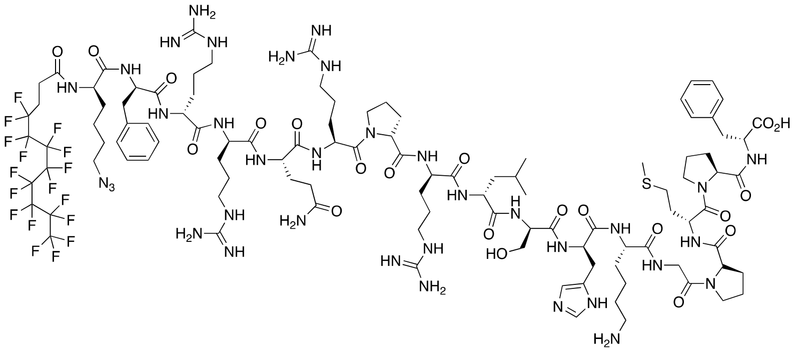

Figure S2: Chemical structure of peptide **4A** [$CF_3(CF_2)_7(CH_2)_2$CO-K(N_3)FRRQRPRLSHKGPMPF-OH]

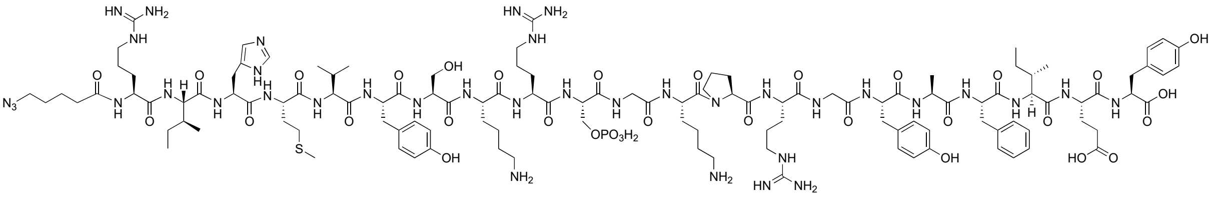

Figure S3: Chemical structure of peptide **5A** [N_3-$(CH_2)_4$-CO-RIHMVYSKR $S^{(PO3H2)}$GKPRGYAFIEY-OH]

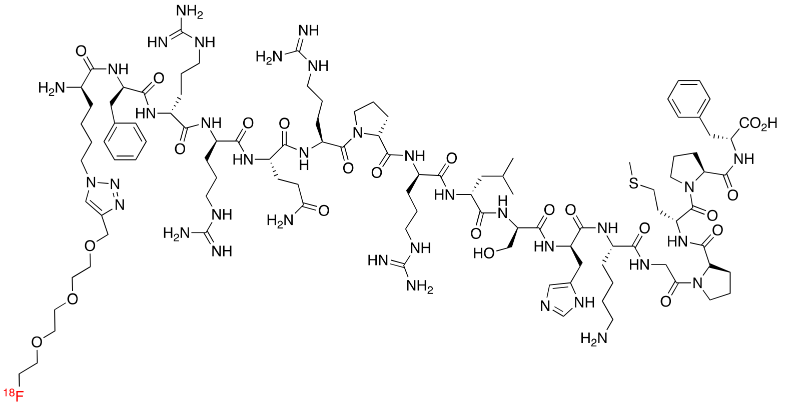

Figure S4: Chemical structure of peptide **[^{18}F]-6A**

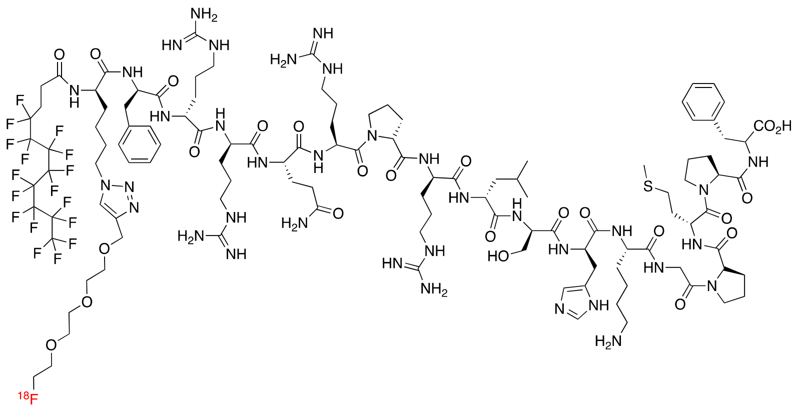

Figure S5: Chemical structure of peptide **[^{18}F]-7A**

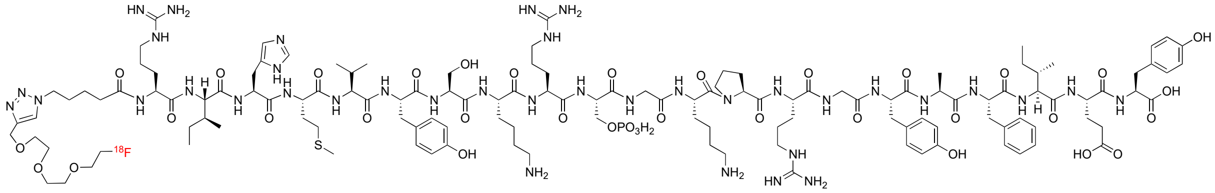

Figure S6: Chemical structure of peptide **[^{18}F]-8A**

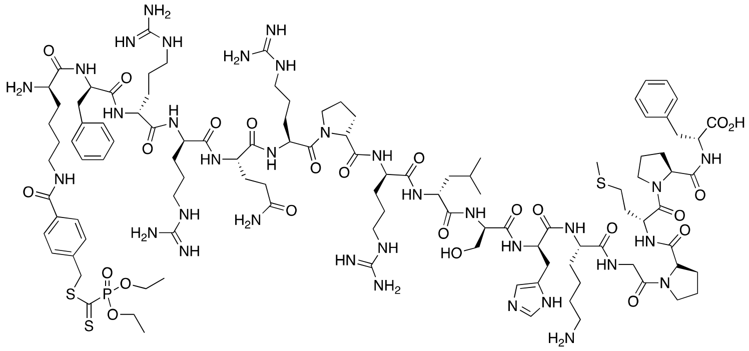

Figure S7: Chemical structure of peptide **3B** [$(Et_2O)P(O)$-C(S)S-CH_2-C_6H_4-CO-KFRRQRPRLSHKGPMPF-OH]

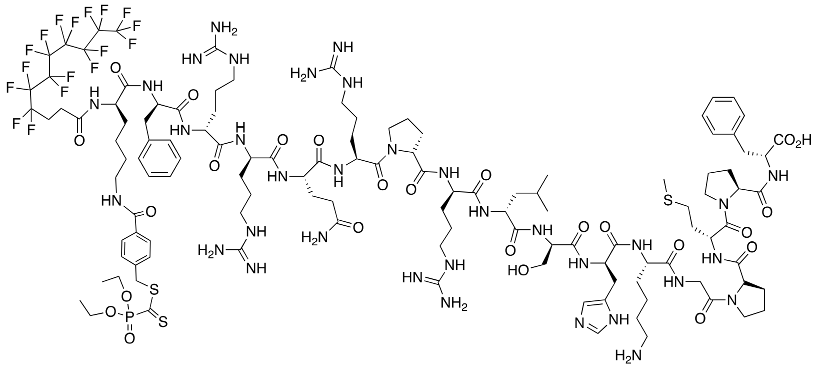

Figure S8: Chemical structure of peptide **4B** [$CF_3(CF_2)_7(CH_2)_2$CO-K($(Et_2O)P(O)$-C(S)S-CH_2-C_6H_4-CO-)FRRQRPRLSHKGPMPF-OH]

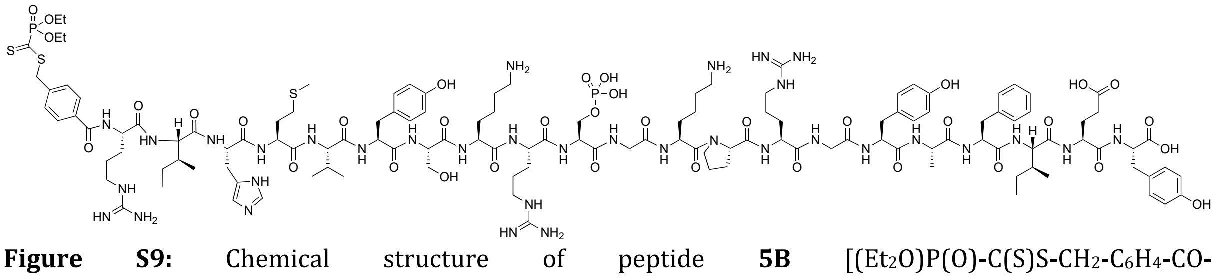

Figure S9: Chemical structure of peptide **5B** [$(Et_2O)P(O)$-C(S)S-CH_2-C_6H_4-CO-RIHMVYSKRS$^{(PO3H2)}$GKPRGYAFIEY-OH]

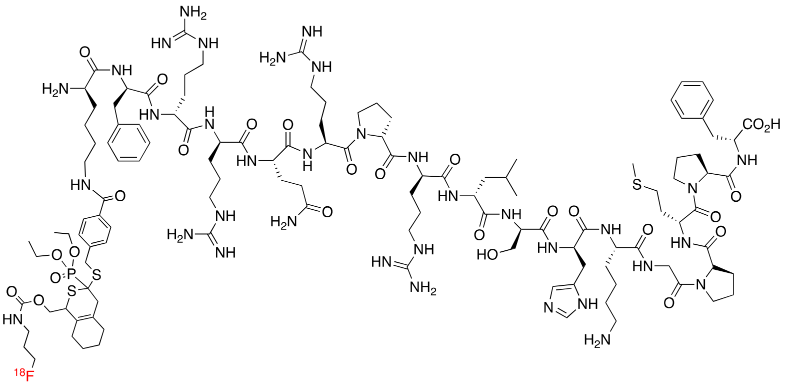

Figure S10: Chemical structure of peptide **[^{18}F]6B**

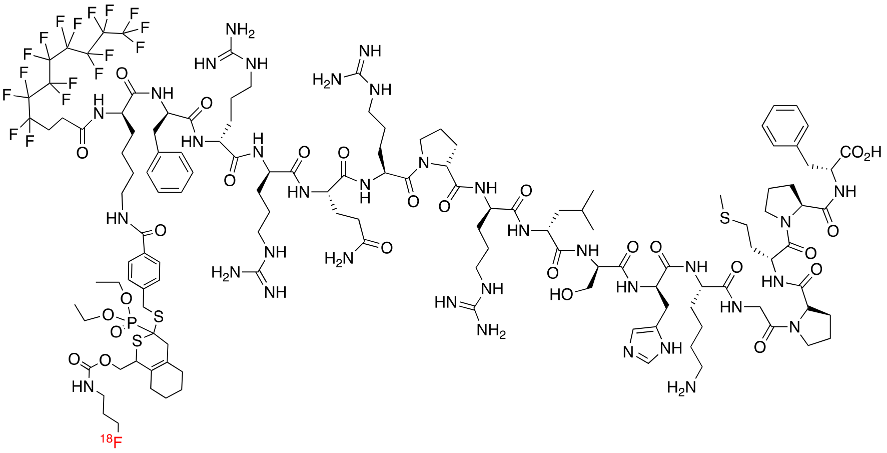

Figure S11: Chemical structure of peptide **[^{18}F]7B**

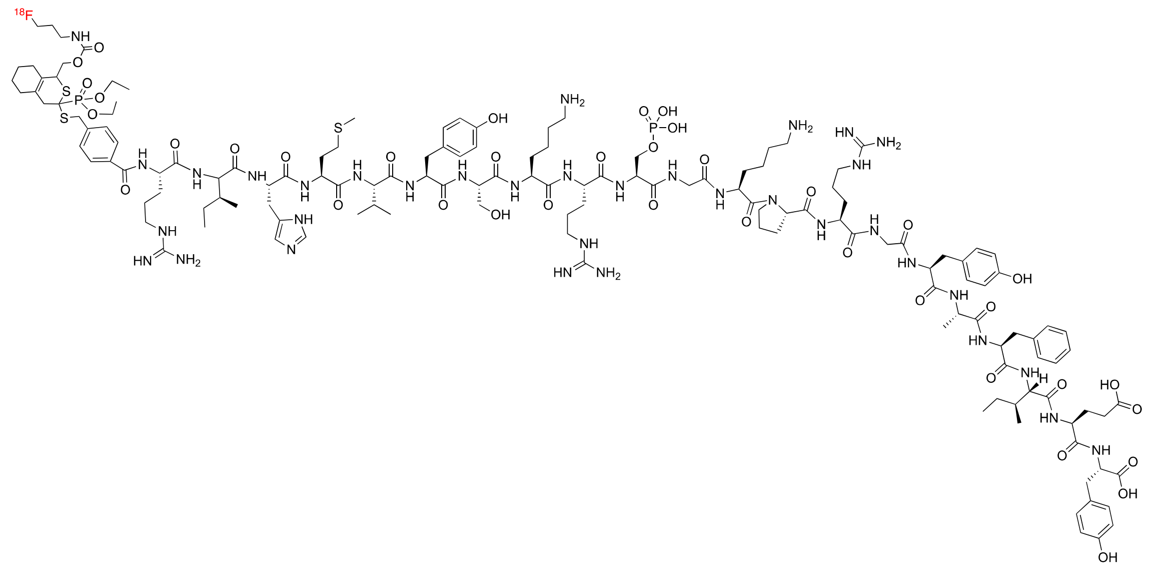

Figure S12: Chemical structure of peptide **[^{18}F]8B**

Generalities (chemistry)

Reagents were obtained from commercial sources and used without any further purification unless otherwise stated otherwise. 4,4,5,5,6,6,7,7,8,8,9,9,10,10,11,11-heptadecafluoroundecanoic acid was purchased from Sigma-Aldrich.
Thin-layer chromatography was performed on Merck silica gel 60F254 plates. VWR silica gel (40-63 µm) was used for chromatography columns. Semi-preparative reverse-phase HPLC purifications were performed on a Waters SunFire C18 OBD Prep column (5 µm, 19 × 150 mm) on a Gilson PLC2020 system. Reverse-phase flash purifications were performed on prepacked Puriflash C18 columns from Interchim on a Gilson PLC2020 system. Analytical reverse-phase HPLC were performed on a Kinetex EVO C18 column (5 µm, 4.6 mm × 150 mm) on an Agilent Technologies 1200 series HPLC system using a linear gradient (5% to 100% v/v in 7.3 min, flow rate of 1.6 $mL.min^{-1}$) of solvent B (0.1% v/v TFA in MeCN) in solvent A (0.1% v/v TFA in H_2O). Preparative HPLC chromatography for purification of peptides were performed with a Gilson PLC2020 system on a SunFire C18 column (5 µm, 19 × 150 mm) using a linear gradient (5% to 95% in 40 min, flow-rate of 18 $mL.min^{-1}$) of solvent B (0.1% TFA in MeCN, v/v) in solvent A (0.1% TFA in H2O, v/v). Detection was set at 220 and 254 nm. Fractions containing the desired products were collected and freeze-dried to give the purified peptides.
Low resolution mass spectra (LRMS) and high resolution mass spectra (HRMS) were obtained on a Shimadzu LCMS 8030 apparatus equipped with a Kinetex C18 column (2.6 µm, 50 X 2.1 mm) using electrospray ionization (ESI) and a triple-quadripole analyzer (TQ) or on an Agilent Technologie 6520 Accurare-Mass Q.Tof LC/MS apparatus equipped with a Zorbax SB C18 column (1.8 µm, 2.1× 50 mm) using electrospray ionization (ESI) and a time-of-flight analyzer (TOF).

Automated SPPS:
The amino acids suitable for a Fmoc/tBu strategy were introduced on a Wang-Phe resin (0.70 $mmol.g^{-1}$) following a classical procedure for manual solid phase peptide synthesis (SPPS). It was performed using a Liberty Blue synthesizer (CEM, France) by standard Fmoc solid-phase chemistry on a preloaded Wang resin (0.67 $mmol.g^{-1}$ resin, 0.1 mmol scale), or on a Rink amide resin (0.42 $mmol.g^{-1}$) using DMF as solvent. The coupling of each amino acid (2M) was carried out using DIC (1M) and Oxyma (1M). Fmoc groups were removed using a 20% v/v solution of piperidine in DMF. Washing steps were performed using DMF. At the end of peptide synthesis, the resin was washed with DCM and MeOH and then dried in DCM.
Manual SPPS:
The amino acids suitable for a Fmoc/tBu strategy were introduced on a rink resin (0.69 $mmol.g^{-1}$) or a Wang-Phe (0.70 $mmol.g^{-1}$) following a classical procedure for manual solid phase peptide synthesis (SPPS). It was performed in polypropylene tubes equipped with polyethylene frits and polypropylene caps using an orbital agitator shaking device. The Fmoc-protected resin was swollen for 1 h in DCM and the excess of solvent was removed by filtration. N-terminal-Fmoc-group was removed by using a 20% (v/v) solution of piperidine in DMF (2 times for 15 min). All Fmoc-deprotection steps were performed in the same way. The piperidine solution was drained off and the resin was washed three times with successively DMF, DCM and MeOH. All Fmoc-protected amino acids (4 equiv.) were coupled in DMF (2.5 mL per 0.1 mmol of resin) for 45 min using HATU (3.8 equiv.) and DIEA (12 equiv.) as activating agents. The excess of solvent was removed by filtration and the resin was washed three times with successively DMF, DCM

and MeOH. With the three amino acids coupled on the resin, N-terminal-Fmoc-group was removed by using a 20% (v/v) solution of piperidine in DMF (2 times for 15 min).

Synthesis of labeling precursors 1A and 1B and non radioactive 2A and 2B

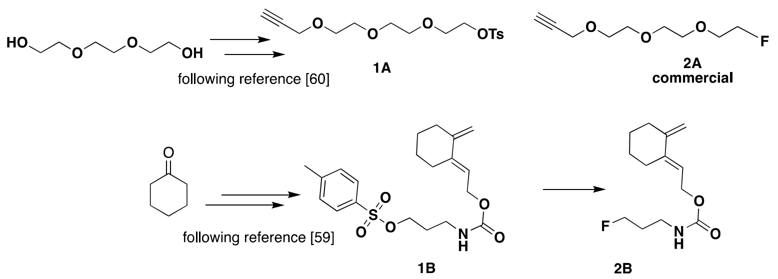

Tosylate **1A** was synthesized according to reference [60]. **2A** was commercially available (ABX, Germany, CAS [1003005-81-9]). **1B** and **2B** were obtained following the described method in reference [59].

Peptide-N_3 synthesis (3A-4A-5A) for CuAAC

Peptides **3A-4A-5A** were synthesized by automated SPPS (see above for the general procedure)

Cleavage and deprotection for peptides (**3A-4A-5A**)

Final peptides (**3A-4A-5A**) were cleaved from the resin under reducing conditions with TFA/H_2O/phenol/thioanisole/EDT 82.5/5/5/5/2.5 (v/v, 1 mL per 0.1 mmol of resin). The mixture was stirred at room temperature for 3 h. The solution was vacuum filtered and the peptides were precipitated with cold diethyl ether (30 volumes per volume of the cleavage mixture). The precipitated peptides were centrifuged at 3000 rpm and 4 °C for 2 min. The precipitate was washed with cold diethyl ether and centrifuged again at 3000 rpm and 4 °C for 2 min. The diethyl ether solution was removed by decantation and the precipitate was dried before HPLC purification.

3A [H-K(N_3)FRRQRPRLSHKGPMPF-OH]

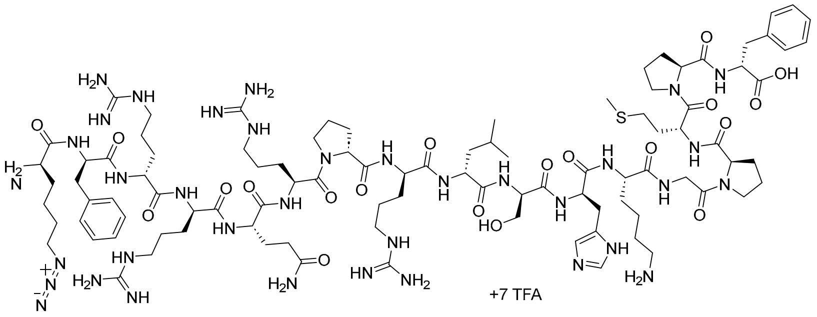

Fmoc-K(N_3)FR(Pbf)R(Pbf)Q(Trt)R(Pbf)PR(Pbf)LSH(Trt)K(Boc)GPMPF-Wang sequence was synthesized following the general method. Finally, the peptide was cleaved under reducing acidic conditions with TFA/H2O/phenol/thioanisole/EDT 82.5/5/5/5/2.5 (v/v) and purified following the general methods affording the title compound as a

white solid (67 mg, 45%). Rt = 3.11 min (>95% purity [220 nm]); MS (ESI) calcd for $C_{96}H_{155}N_{36}O_{20}S$: $[M+H]^+$ 2164.19; found: 2164.19.

4A [$CF_3(CF_2)_7(CH_2)_2CO$-K(N_3)FRRQRPRLSHKGPMPF-OH]

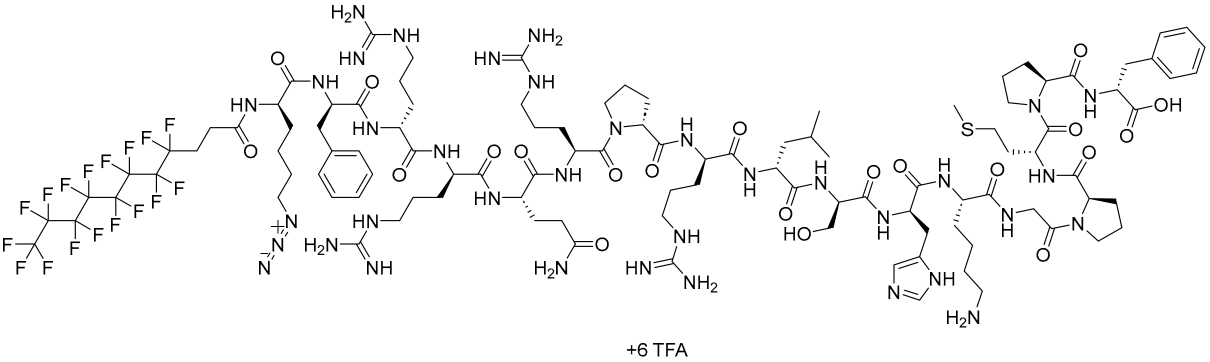

Fmoc-K(N_3)FR(Pbf)R(Pbf)Q(Trt)R(Pbf)PR(Pbf)LSH(Trt)K(Boc)GPMPF-Wang sequence was synthesized following the general method. 4,4,5,5,6,6,7,7,8,8,9,9,10,10,11,11-heptadecafluoroundecanoic acid (2 eq.) was introduced on the resin-bound peptide (100 µmol) in DMF (2 mL per 0.1 mmol of resin) for 45 min using HATU (1.9 eq.) and DIEA (8 eq.) as activating agents. Finally, the peptide was cleaved (TFA/H_2O/phenol/thioanisole/EDT 82.5/5/5/5/2.5 (v/v)) and purified following the general method affording the title compound as a white solid (72 mg, 43%). Rt = 4.70 min (>95% purity [220 nm]); MS (ESI) calcd for $C_{108}H_{160}F_{16}N_{36}O_{21}S$: $[M+H]^+$ 2634.30; found: 2634.30.

5A [N_3-$(CH_2)_4$-CO-RIHMVYSKR $S^{(PO3H2)}$GKPRGYAFIEY-OH]

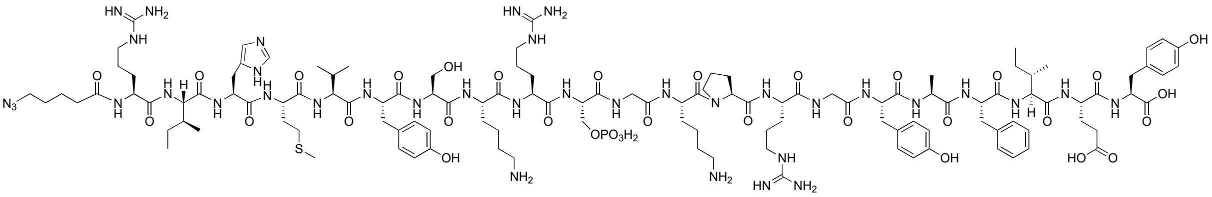

5A was synthesized according to the general procedure. 5-azido-pentanoic acid was coupled on the resin-bound peptide (100 µmol) in DMF (2 mL per 0.1 mmol of resin) for 45 min using HATU (1.9 eq.) and DIEA (8 eq.) as activating agents. After washing, deprotection and purification **5A** was obtained as a white solid (14 mg, 32%). Rt = 10.80 min (94% purity [220 nm]); MS (ESI) calcd for $C_{122}H_{188}N_{37}O_{33}PS$: $[M+H]^+$ 2764.1; found: 2763.33

Non-radioactive reference Peptides

(6A-7A-8A)

Reference peptides were synthesized on small scale (5-10 mg) using a general procedure.

$CuSO_4.5H_2O$ (1.26 mg, 5.06 µmol) and sodium ascorbate (3 mg, 15.2 µmol) in H_2O (165 µL) were mixed and TBTA (2.7 mg, 5.1 µmol) was added quickly (less than 5 minutes after mixing $CuSO_4$ and sodium ascorbate). The solution was added onto the peptide-N_3 (10 mg, 3.37 µmol) and 1-fluoro-2-(2-[2-(prop-2-yn-1-yloxy)ethoxy]ethoxy)ethane (1.28 mg, 6.75 µmol) in DMF (827 µL). The solution was stirred for 3h at 37 °C. After 3h,

EDTA (3eq) was added and the crude solution was purified following the general method affording the title compound as a white solid after freeze drying.

6A

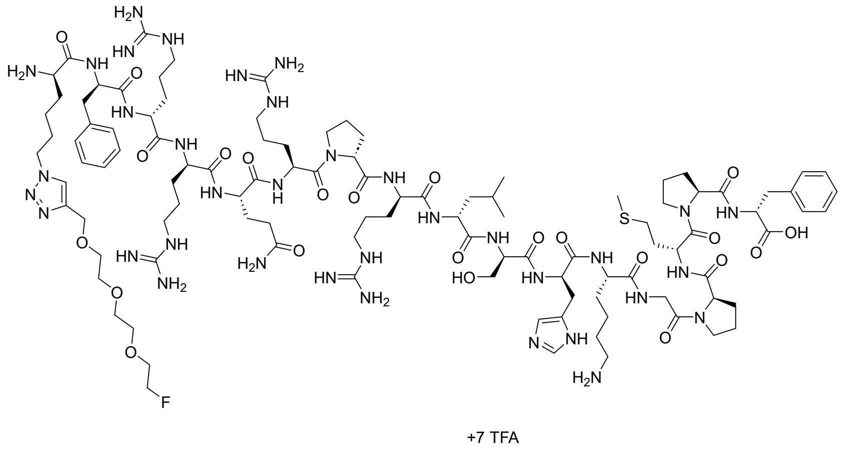

(1 mg, 9%). Rt = 3.76 min (>95% purity [220 nm]); MS (ESI) calcd for $C_{105}H_{169}FN_{36}O_{23}S$: $[M+H]^+$ 2354.29; found: 2354.32

7A

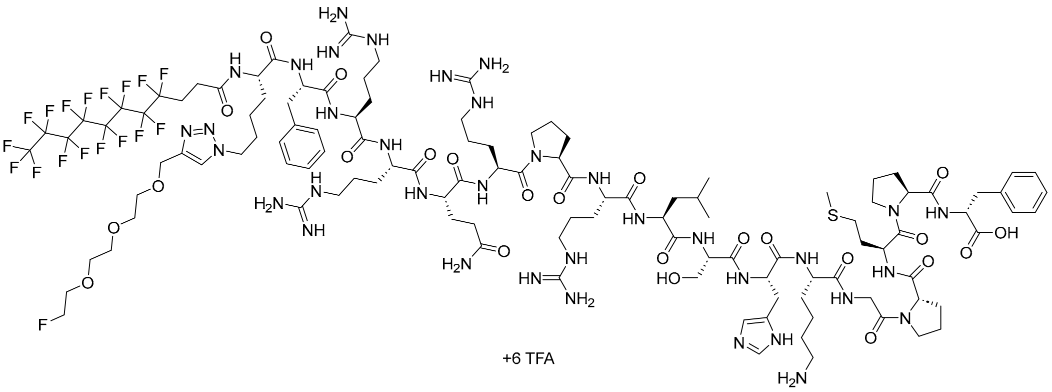

(1.3 mg, 12%). Rt = 6.31 min (>95% purity [220 nm]); MS (ESI) calcd for $C_{116}H_{173}F_{18}N_{36}O_{24}S$: $[M+H]^+$ 2828.28; found: 2828.29

Peptide-dithioesters synthesis (3B-4B-5B) for HDA

General procedure:

Peptides were synthesized according to the general automated procedure and after completion of the sequence the *N*-terminal-Fmoc-group was removed by using a 20% (v/v) solution of piperidine in DMF (2 times for 15 min). After the washing step the resin was transferred in a tube (manual SPPS synthesis procedure) and swollen in DMF.

4-(bromomethyl)benzoic acid (10 equiv.) was dissolved in DCM at 0 °C in a round-bottom flask and a solution of DIC (5 equiv.) in DCM was added. The reaction mixture was stirred 20 min at 0 °C and evaporated under reduced pressure. The resulting slurry was suspended in DMF (3 mL per 0.1 mmol of resin) and transferred in the tube containing the resin. DMAP (0.1 equiv.) was added and the resin was shaken for 1 h. The excess of solvent was removed by filtration and the resin was washed three times with successively DMF, DCM and MeOH and a final DCM wash was performed.

Phosphonodithioformate salt (3 equiv.) was dissolved in DCM (3 mL per 0.1 mmol of resin) and added on the resin which was shaken for 3 h before being filtered and washed as previously.

Peptide cleavage and deprotection

For peptide dithioester **3B**-**4B**-**5B** the deprotection and cleavage from the resin was performed with a solution of TFA/TIS (6 mL per 0.1 mmol of resin, 97/3 v/v).
The solution was filtered and poured in cold Et_2O to precipitate the peptide as a pink solid. This solid was then centrifuged, the Et_2O was discarded and this process was repeated 3 times. The resulting pink solid was purified by semi-preparative reversed-phase HPLC chromatography (Gilson PLC2020 system) on a SunFire C18 column (5 µm, 19 × 150 mm) using a linear gradient (5% to 95% in 40 min, flow-rate of 18 $mL.min^{-1}$) of solvent B (0.1% TFA in MeCN, v/v) in solvent A (0.1% TFA in H2O, v/v). Detection was set at 220 and 254 nm. Fractions containing the desired product were freeze-dried to give the purified phosphonodithioester-peptides (**6B**, **7B**, **8B**) as a pink amorphous solid

3B [(Et_2O)P(O)-C(S)S-CH_2-C_6H_4-CO-KFRRQRPRLSHKGPMPF-OH]

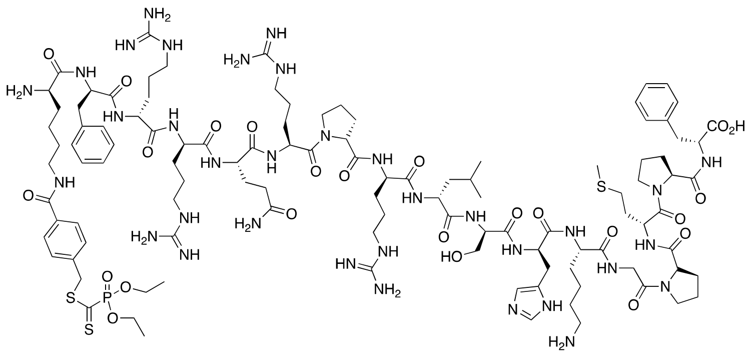

(30.5 mg, 4%). Rt = 5.01 min (>95% purity [220 nm]); MS (ESI) calcd for $C_{109}H_{172}N_{34}O_{24}PS_3$: $[M+H]^+$ 2468.22; found: 2468.22

4B [$CF_3(CF_2)_7(CH_2)_2$CO-K(Et_2O)P(O)-C(S)S-CH_2-C_6H_4-CO-)FRRQRPRLSHKGPMPF-OH]

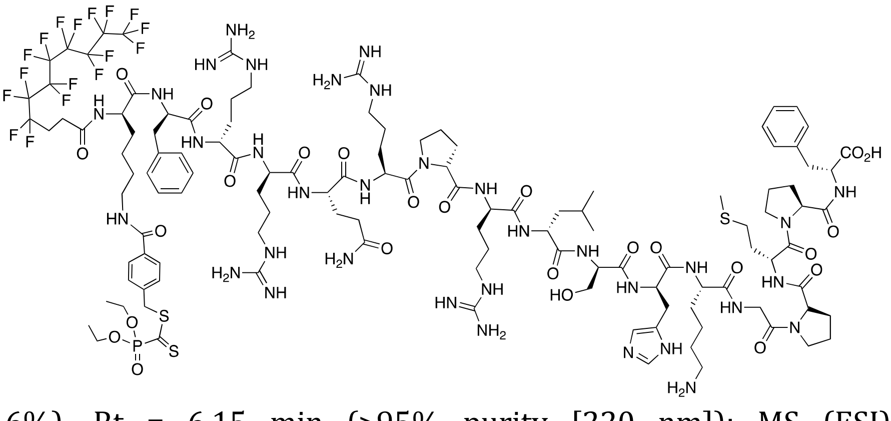

(55.3 mg, 6%). Rt = 6.15 min (>95% purity [220 nm]); MS (ESI) calcd for $C_{120}H_{174}F_{17}N_{34}O_{25}PS_3$: $[M+H]^+$ 2942.21; found: 2942.23

5B [(Et_2O)P(O)-C(S)S-CH_2-C_6H_4-CO-RIHMVYSKR $S^{(PO3H2)}$GKPRGYAFIEY-OH]

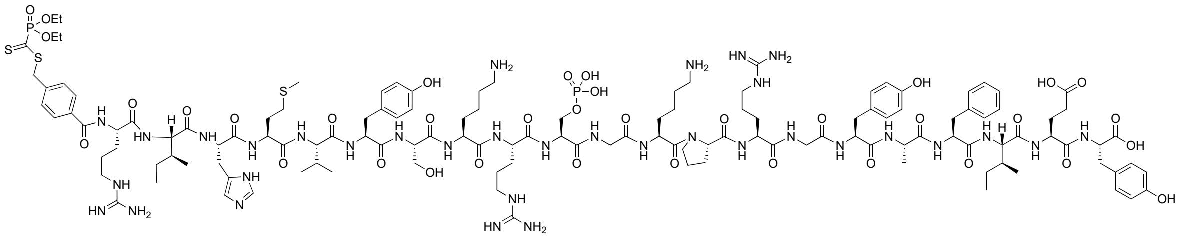

(46 mg, Yield 22%). Rt = 10.25 min (>96% purity [220 nm]); MS (ESI) calcd for $C_{130}H_{196}N_{34}O_{36}P_2S_3$: $[M+H]^+$ 2968.33; found: 2968.32

Non-radioactive reference Peptides

(6B-7B-8B)

General procedure

A solution of peptide-dithioester (**3B**, **4B** or **5B**, 1 equiv.) in water was added to a solution of (E)-2-(2-methylenecyclohexylidene)ethyl (3-fluoropropyl)carbamate (**2B**, 1 equiv.) in iPrOH (total concentration of 0.01 M with a H_2O/iPrOH ratio of 7/3). The reaction mixture was stirred at 60 °C until complete conversion of diene (monitoring by HPLC), cooled to r.t. and concentrated under reduced pressure. The residue was dissolved in DMSO and directly purified by semi-preparative reversed-phase HPLC chromatography (Gilson PLC2020 system) on a SunFire C18 column (5 µm. 19 × 150 mm) using a linear gradient (10% to 95% in 40 min. flow-rate of 18 mL.min^{-1}) of solvent B (0.1% TFA in CH_3CN. v/v) in solvent A (0.1% TFA in H2O. v/v). Detection was set at 220 and 254 nm. Fractions containing the desired products were collected and concentrated under reduced pressure. The residue was freeze-dried to give the purified desired product.

6B

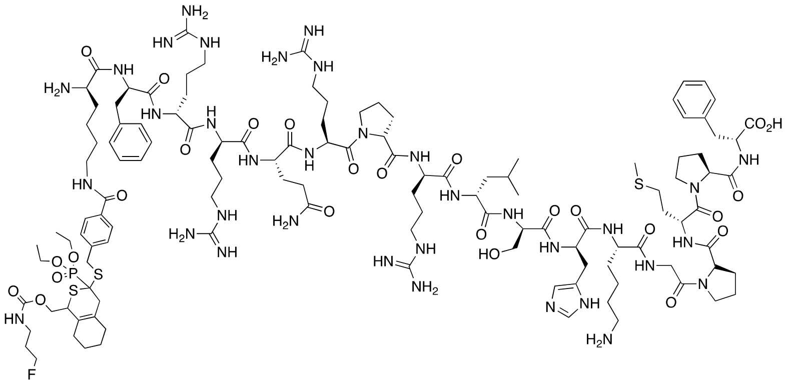

(6 mg, yield 56%). Rt = 5.55 min (>96% purity [220 nm]); MS (ESI) calcd for $C_{122}H_{191}FN_{35}O_{26}PS_3$: $[M+H]^+$ 2709.37; found: 2709.36.

7B

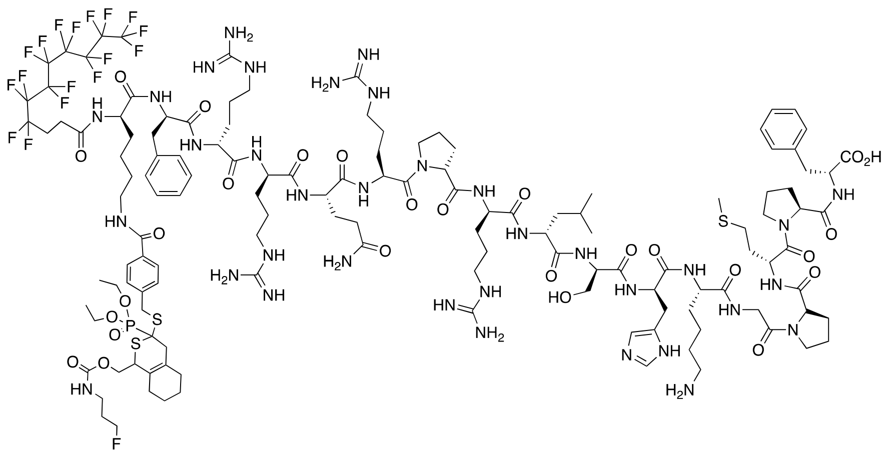

(4.5 mg, yield 36%). Rt = 5.63 min (>95% purity [220 nm]); MS (ESI) calcd for $C_{122}H_{191}FN_{35}O_{26}PS_3$: 637.4777 $(M+5H)^{5+}$; found: 637.4799.

8B

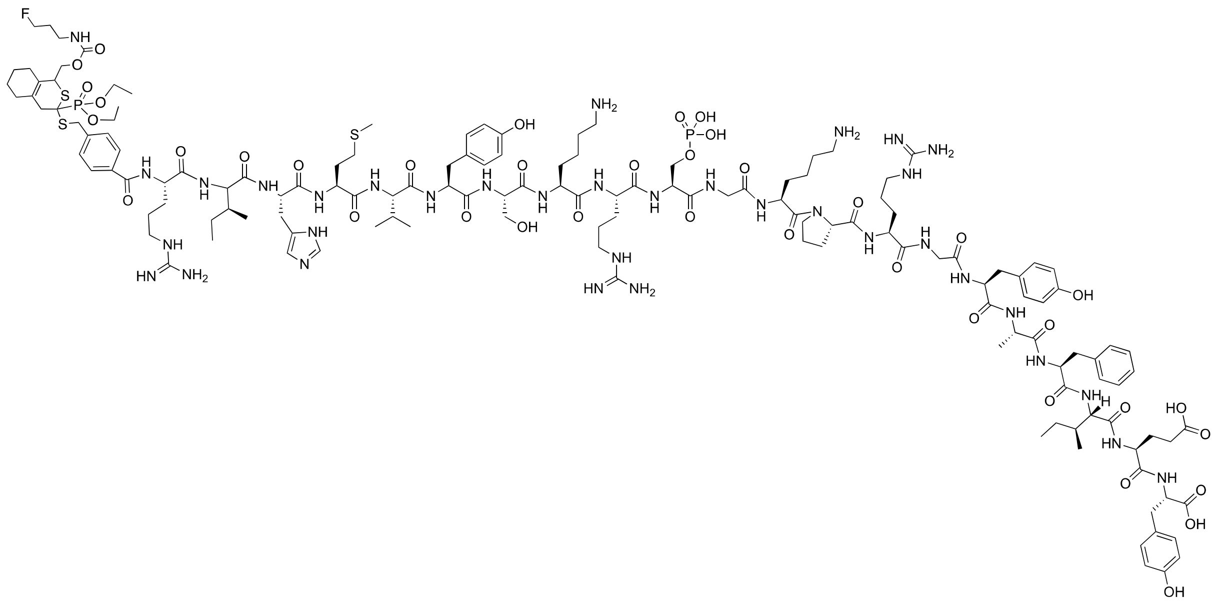

(1.5 mg, yield 19%). Rt = 5.19 min (>95% purity [220 nm]); MS (ESI) calcd for $C_{143}H_{216}FN_{35}O_{38}P_2S_3$: 642.7012 $(M+5H)^{5+}$; found: 642.7023.

RADIOCHEMISTRY

Generalities (radiochemistry)

All reagents for radiochemistry were used without further purification. K_2CO_3 99.99%, Kryptofix $K_{2.2.2}$ (4,7,13,16,21,24-hexaoxa-1,10-diazabicyclo-[8.8.8]-hexacosane), anhydrous acetonitrile 99.8%, ethanol (absolute, HPLC grade), hydrochloric acid, trifluoroacetic acid (HPLC grade), sodium acetate (analytical grade) were purchased from Merck. Accell plus QMA carbonate light cartridges were obtained from Waters (130 mg sorbent, Part N° 186004051) and used as received, C18 Sep-Pak (tC18 environmental WAT 036800 and C18 WAT 020515) were purchased from Waters and pre-conditioned with 5 mL of MeCN or 5 mL of ethanol followed by 10 mL of pure H_2O before use. OASIS HLB Plus short Sep-Pak were obtained from Waters (Part N° 186000132), conditioned with 5 mL of MeCN followed by 10 mL of pure H_2O. $[^{18}O]H_2O$ ($[^{18}O]$ > 98%) for

[^{18}F]fluoride production was purchased from Nukem isotopes GmbH-Germany. Pure H_2O (18.2 MΩ) was produced with a Pure-lab option Q purification system (Veolia). Sodium chloride 0.9% sterile solution was purchased from B BRAUN medical.

[^{18}F]fluoride production was performed with an ACSI 24 MeV cyclotron by proton irradiation of a 1 mL volume niobium target at an energy of 16.5 MeV and an intensity of 35 µA. After 5 min of cooling the radioactivity was transferred to the hot cell under helium pressure then the transfer lines and the target were rinsed twice (2x1 mL) with pure water. Total transferred activity was measured (well counter in the hot cell) and further transferred to the Raytest module reception vial under helium pressure. Residual activity in the intermediate vial after transfer was counted to determine the activity used in each radiosynthesis (a typical activity of 23-24 GBq for 11 µA irradiation was transferred in the hot cell).

Analytical HPLC were performed on a HPLC Dionex U3000 equipped with a DAD detector and a radioactivity detector (NaI) using C18 Kinetex (Phenomenex, 5 µm EVO 4.6x150 mm) or Syncronis (250x4.6 mm 5 µm, Thermo Fisher Scientific) analytical columns with gradient of MeCN/H_2O+0.1% TFA and a 20 µL injection loop. The radioactive detector is placed after the UV-Vis DAD detector generating a delay (function of tubing length and flow rate) between the two signals. The difference observed between the shapes of radioactive signal versus UV-Vis detection is due to the difference in size of the detection cells and the rate of acquisition of the detectors (1 Hz for the radioactive channel versus 5Hz for UV-Vis channel).

PB 0.05M pH 7.5 is a phosphate buffer solution made of $NaH_2PO_4.H_2O$ (1.34g/L), $Na_2HPO_4.7H_2O$ (10.81 g/L) in pure water adjusted to pH 7.5 by dropwise addition of NaOH 1N

Manual synthesis of [^{18}F]2A

56-57 MBq of fluorine-18 in [^{18}O]-H_2O were passed over a QMA cartridge (Waters, 186004051). The cartridge was dried with 10 mL of air and the activity eluted with 1.08 mL of CH_3CN/H_2O (800/280) containing 0.7 mg of K_2CO_3 and 9 mg of $K_{2.2.2}$. The solution was recovered in a 5 mL conical glass vial (Wheaton V-vial) and evaporated under nitrogen flow at 100 °C with iterative addition of CH_3CN (3x1 mL). After full drying, the precursor **1A** (3 mg) in 800 µL of CH_3CN/DMSO (450/350) was added and the vial was sealed and heated at 115 °C during 12 minutes. A sample of the solution was diluted in MeOH/H_2O and injected in HPLC (syncronis column, 250x4.6 mm 5 µm, Thermo Fisher Scientific, H_2O/MeCN 70/30 to 30/70 in 10 minutes, Rt = 8.5 minutes) for analysis. A radiochemical conversion (RCC, radioactive channel) >95% was obtained (Figure S13).

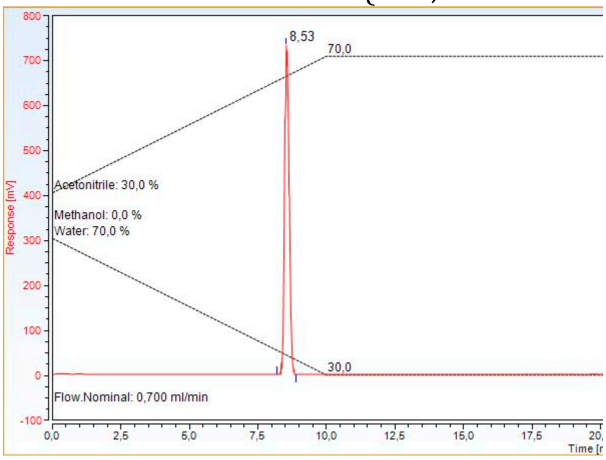

Figure S13: HPLC chromatogram (radioactive channel) of **[^{18}F]2A** obtained by manual synthesis.

Manual synthesis of [^{18}F]2B

20 MBq of fluorine-18 in [^{18}O]-H_2O were passed over a QMA-carbonate cartridge (Waters 186004051). The cartridge was dried with air and the activity was eluted with 1.160 mL of CH_3CN/H_2O (800/360) containing 0.6-0.65 mg of K_2CO_3 and 12-13 mg of $K_{2.2.2}$. The solution was recovered in a 5 mL conical glass vial (Wheaton V-vial) and evaporated under nitrogen flow at 100 °C with iterative addition of CH_3CN (3x1 mL). After full drying, the precursor **1B** (4.5-5 mg) in 1 mL of CH_3CN was added and the vial was sealed and heated at 100 °C during 10 minutes. A sample of the solution was diluted in H_2O and injected in HPLC (column kinetex, phenomenex, 5 µm EVO 4.6x150 mm, H_2O/MeCN 95/5 to 35/65 in 20 minutes, Rt = 11.8 minutes) for analysis. (Figure S14).

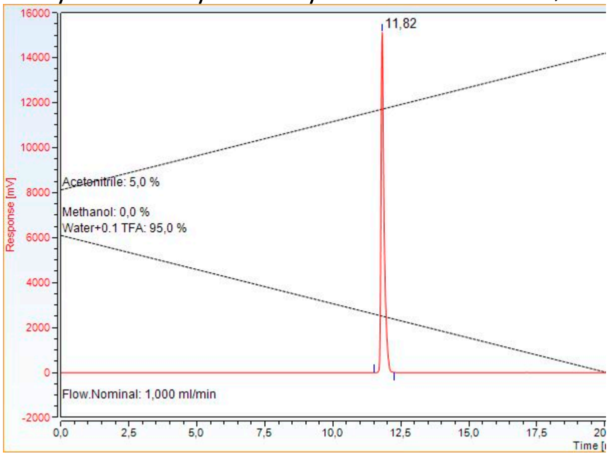

Figure S14: HPLC chromatogram (radioactive channel) of **[^{18}F]2B** obtained by manual synthesis.

Optimization of the CuAAC reactions using peptide 3A

Reaction with THPTA (Main text, table 1 entry 1-3):

Propargylic **[^{18}F]2A** was produced on the SynChrom automated module. After HPLC and formulation on HLB Sep-Pak, **[^{18}F]2A** was recovered in H_2O/ethanol (4/1) for further use in manual reactions. The product was analyzed (Figure S15) by C18 HPLC before use in the following three reactions (syncronis column, 250x4.6 mm 5 µm, Thermo Fisher Scientific, H_2O/MeCN +0.1% TFA 95/5 to 35/65 in 20 minutes).

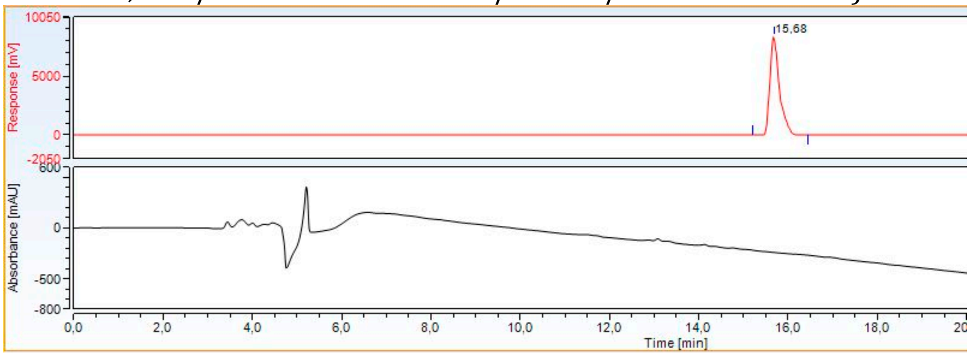

Figure S15: HPLC chromatogram of **[^{18}F]2A**. Top: radioactive channel. Bottom: UV channel220 nm.

For the three reactions described below the same solutions were used:
$CuSO_4.5H_2O$: 0.4 mg/mL (Phosphate buffer, 0.05 M, pH 7.5)
THPTA ((tris(3-hydroxypropyltriazolylmethyl)amine)): 1 mg/mL (Phosphate buffer, 0.05 M, pH 7.5)
Sodium ascorbate: 4 mg/mL (Phosphate buffer, 0.05 M, pH 7.5)
Aminoguanidine.HCl: 4 mg/mL (Phosphate buffer, 0.05 M, pH 7.5)

Peptide-N_3: **3A**, 1mg/mL (H_2O)
[^{18}F]2A: 70 µL (H_2O/ethanol 4/1)
All reactions were analyzed by HPLC (syncronis column, 250x4.6 mm 5 µm, Thermo Fisher Scientific, H_2O/MeCN +0.1% TFA 95/5 to 35/65 in 20 minutes).

Reaction 1 (Table 1 in main text, entry 1)
In a 2mL eppendorf tube were introduced the peptide **3A** (0.01 mg, 3.3 nmol, 10 µL), 70 µL of **[^{18}F]2A** (31 MBq) and aminoguanidine (0.22 mg, 2 µmol, 55 µL). A solution of $CuSO_4$ (0.01 mg, 0.04 µmol, 25 µL), THPTA (0.09 mg, 0.2 µmol, 90 µL) and sodium ascorbate (0.4 mg, 2 µmol, 100 µL) was quickly prepared (less than 5 minutes) and added to the previous solution, the volume was adjusted to 700 µL by addition of 350 µL of phosphate buffer (pH 7.5, 0.05M). The solution was stirred and heated in a water bath at 37-38 °C during 30 minutes. The solution was treated with 1 mL of EDTA (20 mg/mL) and injected into analytical HPLC (figure S16, top).

Reaction 2 (Table 1 in main text, entry 2). The same protocol was used for reaction N°2 but **without peptide**.
In a 2mL eppendorf tube was introduced 70 µL (25 MBq) of **[^{18}F]2A** and aminoguanidine (0.22 mg, 2 µmol, 55 µL). A solution of $CuSO_4$ (0.01 mg, 0.04 µmol, 25 µL), THPTA (0.09 mg, 0.2 µmol, 90 µL) and sodium ascorbate (0.4 mg, 2 µmol, 100 µL) was quickly prepared (less than 5 minutes) and added to the previous solution, the volume was adjusted to 700 µL by addition of 360 µL of phosphate buffer (pH 7.5, 0.05M). The solution was stirred and heated in a water bath at 37-38 °C during 30 minutes. The solution was treated with 1 mL of EDTA (20 mg/mL) and injected into analytical HPLC (figure S16, middle).

Reaction 3 (Table 1 in main text, entry 3). The same protocol was used for reaction N°3 but **without peptide** and **without THPTA**.
In a 2mL eppendorf tube was introduced 70 µL (12 MBq) of **[^{18}F]2A** and aminoguanidine (0.22 mg, 2 µmol, 55 µL). A solution of $CuSO_4$ (0.01 mg, 0.04 µmol, 25 µL) and sodium ascorbate (0.4 mg, 2 µmol, 100 µL) was prepared and quickly added to the previous solution, the volume was adjusted to 700 µL by addition of 450 µL of phosphate buffer (pH 7.5, 0.05M). The solution was stirred and heated in a water bath at 37-38 °C during 30 minutes. The solution was treated with 1 mL of EDTA (20 mg/mL) and injected into analytical HPLC (figure S16, bottom).

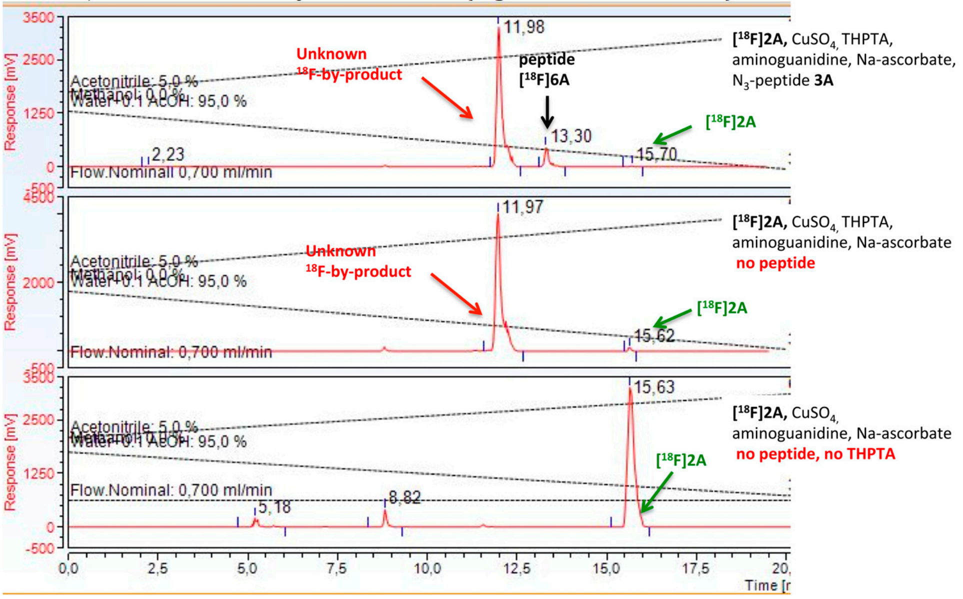

Figure S16: HPLC chromatograms (radioactive channel) of reaction 1 with THPTA (top), reaction 2 with THPTA in absence of peptide (middle) and reaction 3 without THPTA and without peptide (bottom): syncronis column, 250x4.6 mm 5 µm, Thermo Fisher Scientific, H_2O/MeCN +0.1% TFA 95/5 to 35/65 in 20 minutes, flow 0.7 mL/min.

Optimized conditions of the CuAAC reaction (manual) with peptide 3A.

$CuSO_4$: 5 mg/mL (H_2O)
Sodium ascorbate: 30 mg/mL (Phosphate buffer, 0.05 M, pH 7.5)
Aminoguanidine.HCl: 5.4 mg/mL (Phosphate buffer, 0.05 M, pH 7.5)
Peptide-N_3: **3A**, 1mg/mL (H_2O)

In a 2mL eppendorf tube were introduced 70 µL (65 MBq) of **[^{18}F]2A**, peptide **3A** (75 µL, 75 µg, 25 nmol) and aminoguanidine (1.68 mg, 15 µmol, 315 µL). A solution of $CuSO_4$ (0.5 mg, 3.1 µmol, 100 µL) and sodium ascorbate (3 mg, 15 µmol, 100 µL) was quickly prepared and added to the previous solution, the volume was adjusted to 700 µL by addition of 40 µL of phosphate buffer (pH 7.5, 0.05M). The solution was stirred and heated in a water bath at 40 °C during 30 minutes. At 15 and 30 minutes a sample of the solution was treated with 1 mL of EDTA (20 mg/mL) and injected into analytical HPLC (figure S17, top at 15 minutes, bottom after 30 minutes of reaction)

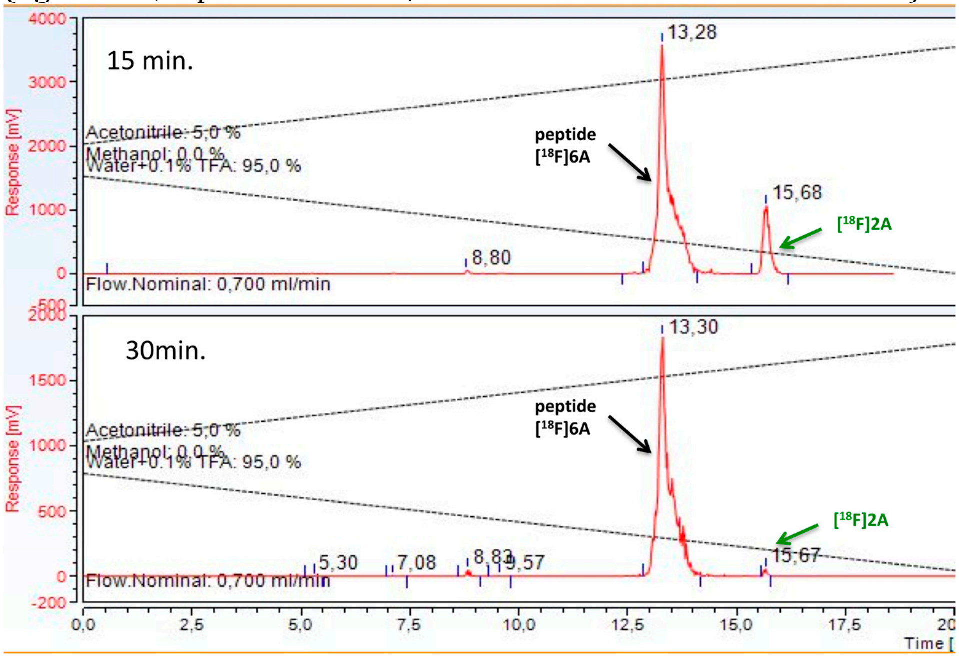

Figure S17: HPLC chromatogram (radioactive channel) of **[^{18}F]6A** (Top: after 15 minutes of reaction; Bottom after 30 minutes of reaction). Syncronis column, 250x4.6 mm 5 µm, Thermo Fisher Scientific, H_2O/MeCN +0.1% TFA 95/5 to 35/65 in 20 minutes, flow 0.7 mL/min. **[^{18}F]6A** Rt = 13.3 and **[^{18}F]2A** Rt = 15.6 minutes.

Optimized conditions of the HDA reaction with peptides 4B and 5B

A batch of **[^{18}F]2B** (in pure ethanol) was produced on a Synchrom (Raytest) EVO III module. The [^{18}F]-diene was purified by HPLC and the collected fraction was adsorbed on two C18 Sep-Paks. After drying, **[^{18}F]2B** was eluted with 5 mL of pure ethanol (1GBq/mL). The product was analyzed by C18 HPLC (column C18 Kinetex Phenomenex, 5 µm EVO 4.6x150 mm, H_2O/MeCN + 0.1 % TFA 95/5 to 0/100 in 7 minutes, Rt = 7.23 minutes, purity > 96%, Figure S18).

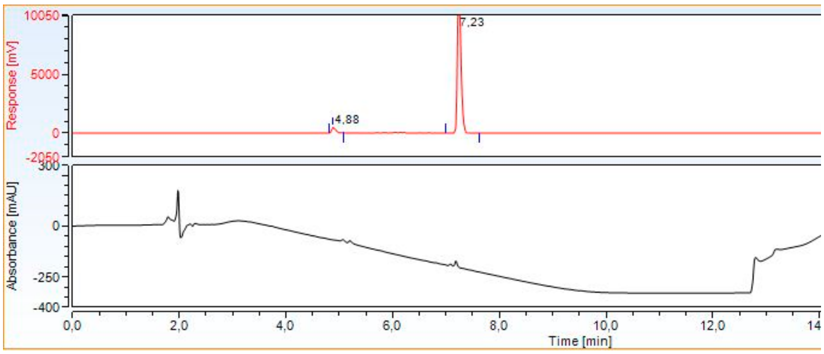

Figure S18: HPLC chromatogram of **[18F]2B.** Top: radioactive channel. Bottom: UV channel 220 nm.

HDA reaction of 5B in glass reactor

In a 4 mL glass tube were mixed **[18F]2B** (in 10 µL of ethanol) and peptide-dithioester **5B** (4.5 µg, 1.2 nmol in 40 µL of H_2O). The solution was heated at 62-63 °C during 30 minutes. The solution was analyzed by HPLC after 30 minutes of reaction (column C18 Kinetex Phenomenex, 5 µm EVO 4.6x150 mm, H_2O/MeCN + 0.1 % TFA 95/5 to 0/100 in 7 minutes, figure S19).

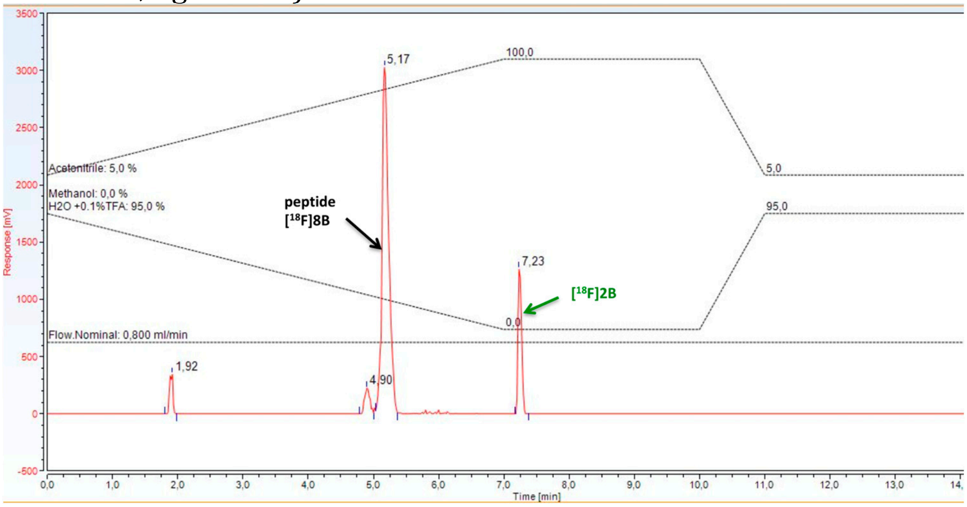

Figure S19: HPLC chromatogram (radioactive channel) of **[18F]8B** (manual reaction)

HDA reaction of 4B in glass reactor

In a 4 mL glass tube were mixed **[18F]2B** (in 10 µL of ethanol) and peptide-dithioester **4B** (6 µg, 1.6 nmol in 40 µL of H_2O). The solution was heated at 62-63 °C during 45 minutes. The solution was analyzed by HPLC after 30 minutes of reaction (column C18 Kinetex Phenomenex, 5 µm EVO 4.6x150 mm, H_2O/MeCN + 0.1 % TFA 95/5 to 0/100 in 7 minutes, figure S20 bottom).

HDA reaction of 4B in polypropylene reactor

In a 1.5 mL polypropylene cryo-tube were mixed **[18F]2B** (in 10 µL of ethanol) and peptide-dithioester **4B** (6 µg, 1.6 nmol in 40 µL of H_2O). The solution was heated at 62-63 °C during 30 minutes. The solution was analyzed by HPLC after 30 minutes of reaction (column C18 Kinetex Phenomenex, 5 µm EVO 4.6x150 mm, H_2O/MeCN + 0.1 % TFA 95/5 to 0/100 in 7 minutes, figure S20 top).

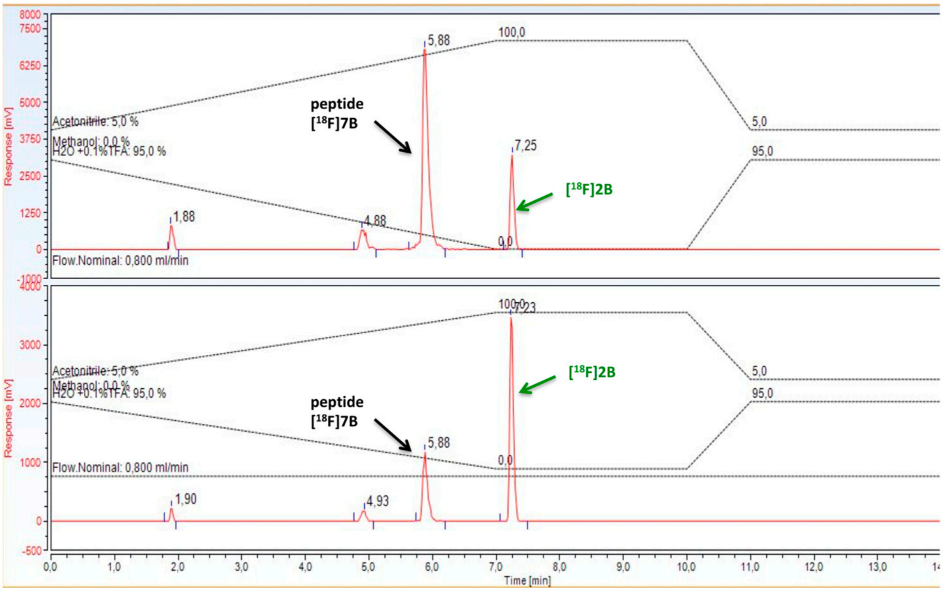

Figure S20: HPLC chromatogram (radioactive channel) of **[^{18}F]7B** (top: reaction in polypropylene reactor, bottom: reaction in glass reactor)

Automated Synthesis

Generalities

A Raytest R&D SynChrom (Raytest) EVO III dual reactor was used for the automated radiosyntheses. Outlet of the pump and vents are connected to gas bags to avoid any radioactive releases in the hot cell ventilation system, 3 additional valves (G3-G5) and a 12 mL vial were added to measure the ^{18}F activity transferred from the target. The system is equipped with a semi preparative HPLC (Knauer) including an isocratic pump, a 254 nm fixed wavelength UV detector, a radioactivity detector and a 5 mL stainless steel injection loop.
After irradiation of [^{18}O]H_2O, the radioactivity is transferred into the hot cell under helium pressure then the transfer lines and the target are rinsed twice (2x1 mL) with pure water. Total transferred activity is measured (well counter in the hot cell, **1**) and further transferred to the Raytest module reception vial (**2**, figure S7 and figure S9) under helium pressure. Residual activity in the intermediate vial after transfer is counted to determine the activity used in each radiosynthesis (a typical activity of 23 GBq for 11 µA irradiation was transferred in the hot cell).

Fully automated CuAAC on peptides 3A, 4A, 5A.

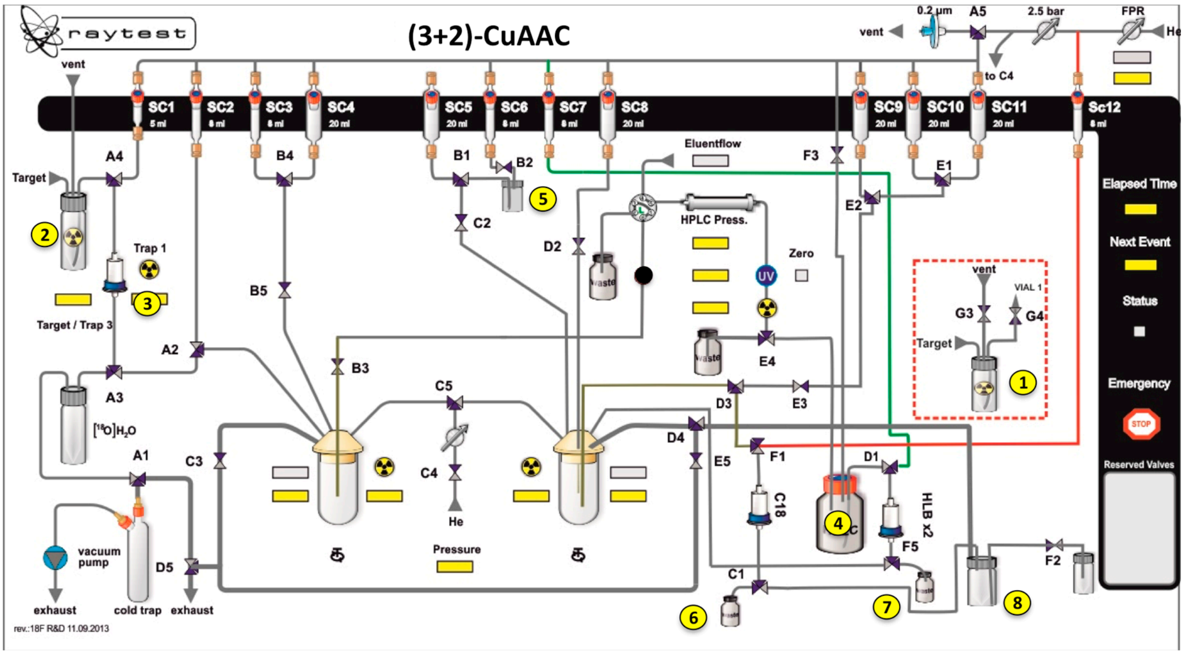

Scheme S1: Layout of the **automated CuAAC** process on SynChrom EVOIII module.
Reagent list:

SC1: K_2CO_3 0.7, $K_{2.2.2}$ 10 mg, CH_3CN 800 µL, H_2O 280 µL
SC2: CH_3CN 1.7 mL
SC3: **1A** (3 mg) CH_3CN/DMSO 450/350 µL
SC4: H_2O 4.4 mL
SC5: **3A-5A** 0.3 mg, 0.45 mL H_2O/DMF, 90/10, aminoguanidine 3.6 mg, Na gentisate, 2 mg, H_2O 0.25 mL
SC6: Na ascorbate 6.3 mg, 0.3 mL of H_2O
SC7: Acetone 1.6 mL
SC8: EDTA 20 mg, 8 mL H_2O
SC9: Diethyl ether 3 mL
SC10: H_2O/ethanol (80/20) 7 mL
SC11: ethanol 1.1 mL, HCl 0.2M 0.2 mL
SC12: H_2O 4 mL
Vial **5**: $CuSO_4.5H_2O$ 0.8 mg, 0.1 mL H_2O
Dilution bottle **4**: H_2O 25 mL
Formulation and collect **8**: Na gentisate (80 mg), methionine (15 mg), H_2O 7 mL

A Raytest R&D SynChrom dual reactor was used for the automated radiosynthesis with some modifications. Reactor 1 is used for the synthesis of the prosthetic group (**[^{18}F]2A**) and the second reactor is used for the CuAAC reaction.
For the synthesis of **[^{18}F]6A-[^{18}F]8A** a separated 3 mL V-vial (**5**) was added between **SC6** and the reactor 2 to separate the $CuSO_4$ from the sodium ascorbate and to mix them at the last moment only (scheme S1).
The activity (22 +/- 2 0 GBq) received in the intermediate vial (**1**) is transferred onto the automated module (**2**), and then trapped on a QMA-carbonate cartridge (**3**, Waters Sep-Pak Light Accell plus QMA, 186004051). After drying, the [^{18}F]fluoride is eluted into the reactor 1 with a solution of $K_{2.2.2}$ (10 mg in 800 µl of MeCN) and K_2CO_3 (0.7 mg in 280 µl of water, **SC 1**). The solution is evaporated under reduced pressure and argon flow at 90 °C with iterative additions of MeCN (3 times, total volume used 1.7 mL, **SC2**). After evaporation, tosylate **1A** (3 mg, 8.7 µmol, **SC3**) in 800 µL of MeCN/DMSO (450/350 v/v) is added and the sealed reactor is heated at 115 °C during 10 min. After cooling to 45 °C, the crude solution is diluted with H_2O (4.4 mL, **SC4**). The whole solution is injected in HPLC (5 mL injection loop) and purified on a syncronis C18 column (Thermo scientific, 250x10 mm, 5 µm) using MeCN/H_2O (80/20 v/v) at a flow rate of 5 mL/min (conversion according to semi-preparative HPLC on radioactive channel > 90%, Rt = 15', chromatogram Figure S21).
The collected solution (< 13 mL) is diluted in 25 mL of water (**4**) and passed through two stacked HLB cartridges (Waters 186000132 and 186005125, pre-conditioned with MeCN 5mL and H_2O 10 mL). The cartridges are then dried, washed with 4 mL of H_2O (**SC12**). **[^{18}F]2A** is then eluted into reactor 2 with 1.6 mL of acetone (**SC7**). Acetone is

evaporated without drying (5 minutes) until the temperature of the reactor reaches 9-10 °C. Content of **SC5** is then added (Peptide-N_3 **3A-5A** 0.3 mg, 0.1 µmol in H_2O/DMF, 90/10, 0.45 mL, aminoguanidine 3.6 mg, sodium gentisate, 2 mg in 0.25 mL H_2O) in reactor 2. The $CuSO_4.5H_2O$ (0.8 mg, 3.2 µmol, in 0.1 mL H_2O; intermediate vial **5**) and sodium ascorbate (**SC6**, 6.3 mg, 31.5 µmol in 0.3 mL of H_2O) are then added and the reactor is heated 30 minutes at 55 °C.
After cooling, the crude solution is diluted with a solution of EDTA (20 mg in 8 mL of H_2O, **SC8**) and transferred under pressure on the C18 cartridge (WAT 036805 pre-conditioned with 5 mL of EtOH and 10 mL of H_2O). The cartridge is dried and washed with 3 mL of diethyl ether (**SC9**) to remove unreacted **[^{18}F]2A**. The cartridge is dried under argon flow during 2 minutes and then washed with 7 mL of H_2O/ethanol (80/20, **SC10**). After a brief drying under argon the peptides **[^{18}F]6A-8A** are eluted with an acidic solution of ethanol (1.1 mL of ethanol containing 0.2 mL of HCl 0.2M, **SC11**). To ensure a good stability, [^{18}F]-peptides were formulated in the collect vial (**8**) with a solution of sodium gentisate (80 mg) and methionine (15 mg) in 7 mL of water for injection.

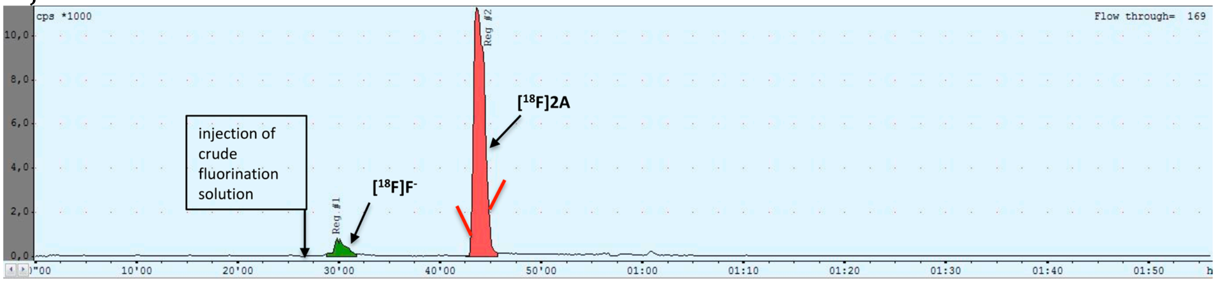

Figure S21: typical semi-preparative HPLC chromatogram (radioactive channel only) of **[^{18}F]2A** on fully automated radiosynthesis. Red lines indicate the start and end of the collect of **[^{18}F]2A** (maximum 13 mL of solution collected).

Fully automated HDA on peptides 3B, 4B, 5B.

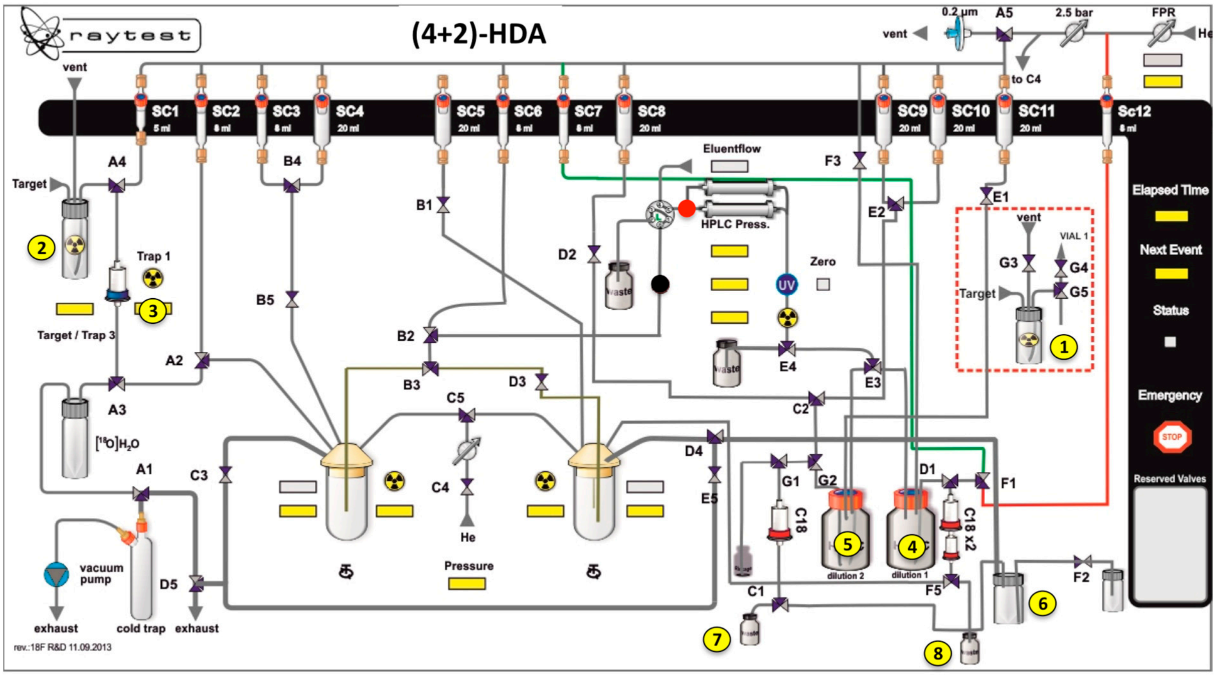

Scheme S2: Layout of the **automated HDA** process on SynChrom EVOIII module.
Reagent list:

SC1: K_2CO_3 0.6 mg, $K_{2.2.2}$ 13 mg, CH_3CN 800 µL, H_2O 360 µL
SC2: CH_3CN 1.7 mL
SC3: **1B** (5 mg) CH_3CN 1mL
SC4: H_2O 2.2 mL CH_3CN 2 mL

SC5: **3B-5B** 3 mg, 3.75 mL H_2O
SC6: CH_3CN 6 mL
SC7: Ethanol 1.45 mL
SC8: 5 mL H_2O
SC9: Ethanol 1.15 mL, HCl 0.2M 30 µL
SC10: NaCl 0.9% 9 mL
SC11: Empty
SC12: Ethanol 0.45 mL
Dilution bottle **4**: H_2O 60 mL
Dilution bottle **5:** H_2O 30 mL
Collect **6**: 15 mL empty vial

A Raytest R&D SynChrom (Raytest) dual reactor was used for the automated radiosynthesis with some modifications. Reactor 1 is used for the synthesis of the prosthetic group (**[^{18}F]2B**) and the second reactor is used for the HDA reaction (scheme S2).
For the synthesis of **[^{18}F]6B-[^{18}F]8B** a second HPLC purification on a semi-preparative Kinetex column (Phenomenex) was necessary. An USB remotely controlled 6 ways-2 positions Titan MX II valve (Rheodyne) was inserted as a column selector (red circle on scheme S2) enabling to shift between the two different columns. Injection loop and tubing common for the two purifications processes are washed automatically with MeCN after use (6 mL from **SC6**). For the purification of **[^{18}F]6B-[^{18}F]8B** a gradient was obtained by adding an automated injection pump (KdScientific) performing a direct injection of MeCN in the solvent bottle with constant stirring. A second dilution flask (50 mL falcon tube, **5**) was added for the dilution of the final product in water before C18 Sep-Pak formulation.
The activity (22.4-23.0 GBq) received in the intermediate vial is transferred onto the automated module and then trapped on a QMA cartridge (**3**, Waters Sep-Pak Light Accell plus QMA, 186004051). After drying, the [^{18}F]fluoride is eluted into reactor 1 with a solution of $K_{2.2.2}$ (13-14 mg in 800 µl of MeCN) and K_2CO_3 (0,6 mg in 360 µl of water, **SC 1**). The solution is evaporated under reduced pressure and argon flow at 90 °C with iterative additions of MeCN (3 times, total volume used 1.7 mL, **SC2**). After evaporation, tosylate **1B** (5.0 mg, 12 µmol, **SC3**) in 1 mL of MeCN is added and the sealed reactor is heated at 95 °C for 10 min. After cooling to 45 °C, the crude solution is diluted with H_2O (2.2 mL) and MeCN (2 mL, **SC4**). The whole solution is injected in HPLC (5 mL injection loop) and purified on a Syncronis C18 column (Thermo scientific, 250x10 mm, 5 µm) using MeCN/H_2O (60/40 v/v) at a flow rate of 4 mL/min (conversion according to semi-preparative HPLC on radioactive channel > 90%, Rt = 14.5 minutes, figure S22 left part).
The collected solution (12 mL) is diluted in 60 mL of water (**4**) and passed through two stacked C18 cartridges (tC18 environmental WAT 036800 and C18 WAT 020515, WATERS). The cartridges are then dried and washed with 0.45 mL of ethanol (**SC12**), **[^{18}F]2B** is eluted into reactor 2 with 1.45 mL of alcohol (EtOH HPLC grade, **SC7**).
Peptide-dithioester **3B-5B** (3 mg, 0.8 µmol) in 3.75 mL of H_2O (**SC5**) are added and the solution is stirred and heated 45 min at 62-65 °C. During the click reaction, the injection loop and transfer lines to HPLC are washed with 6 mL of MeCN (**SC6**). After cooling at 50 °C the solution is injected in HPLC (5 mL injection loop) and purified on a Kinetex EVO C18 column (Thermo scientific, 150x10 mm, 5 µm) using H_2O/MeCN + 0.1% TFA at a flow rate of 4 mL/min with an increasing gradient of MeCN (figure S22, right part). The purified product is collected and diluted in 30 mL of water (**5**) and passed over a C18 cartridge (C18 WAT 020515, Waters). The cartridge is washed with 5 mL of water to remove solvents and TFA (**SC8**) and then dried before elution with 1.15 mL of ethanol (spiked with 30 µL of HCl 0.2M, **SC9**). The peptides are further diluted with NaCl 0.9% (9 mL passed over the C18 Sep-Pak cartridge, **SC10**) and collected into the final vial **6**.

HPLC parameters for purification of peptides **[^{18}F]6B-8B**:

[^{18}F]6B flow 4 mL.min^{-1} during 7 minutes (H_2O/MeCN 90/10 +0.1% TFA, 50 mL) then addition of MeCN 100% at a flow of 4 mL.min^{-1} in the solvent bottle. Rt = 17 minutes
[^{18}F]7B flow 4 mL.min^{-1} during 7 minutes (H_2O/MeCN 95/5 +0.1% TFA, 50 mL) then addition of MeCN 100% at a flow of 3 mL.min^{-1} in the solvent bottle. Rt = 16 minutes
[^{18}F]8B flow 4 mL.min^{-1} during 7 minutes (H_2O/MeCN 95/5 +0.1% TFA, 100 mL) then addition of MeCN 100% at a flow of 3 mL.min^{-1} in the solvent bottle. Rt = 17.8 minutes

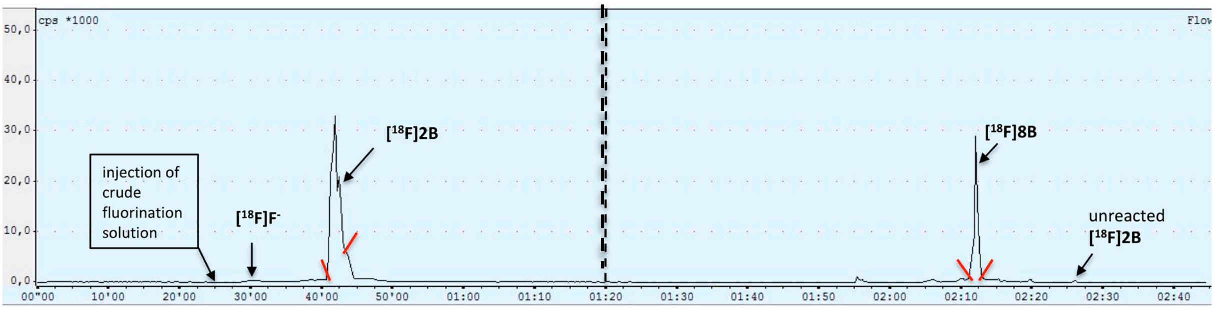

Figure S22: typical radioactive HPLC chromatogram (semi-preparative) of fully automated HDA reaction on module. Red lines indicate the start and end of collect of the products. Left part: purification of **[^{18}F]2B**. Right part: purification of **[^{18}F]8B** after the HDA reaction.

HPLC analyses of purified peptides and co-injection with non radioactive references

HPLC analyses of peptides [^{18}F]6A-8A (CuAAC)

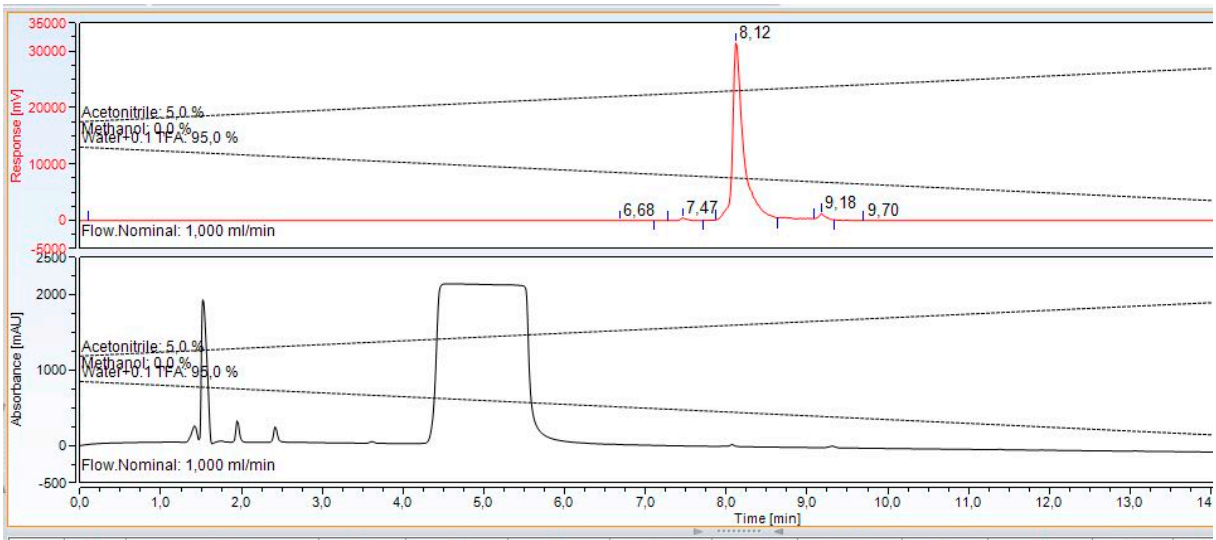

	A	B	C	D	E	F	G	H	I	J	K	L
1	Peak	Peak Name	Ret.Time	Rel.Area	Area	Height	Match	RSD Match	PPI	RSD PPI		
3	No.		min	%	mV*min	mV		%	nm	%		
4	1		6,683	0,00	0,2058	4,91	n.a.	n.a.	n.a.	n.a.		
5	2		7,467	0,73	36,3150	416,58	n.a.	n.a.	n.a.	n.a.		
6	3		8,117	97,36	4812,8713	31225,70	n.a.	n.a.	n.a.	n.a.		
7	4		9,183	1,62	80,2384	961,93	n.a.	n.a.	n.a.	n.a.		
8	5		9,700	0,28	13,6063	0,00	n.a.	n.a.	n.a.	n.a.		

Figure S23: Analytical HPLC of purified and formulated **[^{18}F]6A** (top: radioactive channel, bottom: UV channel 215 nm). Integration table refers to the radioactive channel. The broad and intense signal in UV is due to the sodium gentisate (80 mg/mL). Column Kinetex (phenomenex) H_2O/CH_3CN + 0.1% TFA 95/5 to 35/65 in 20 minutes at a flow rate of 1 mL/min.

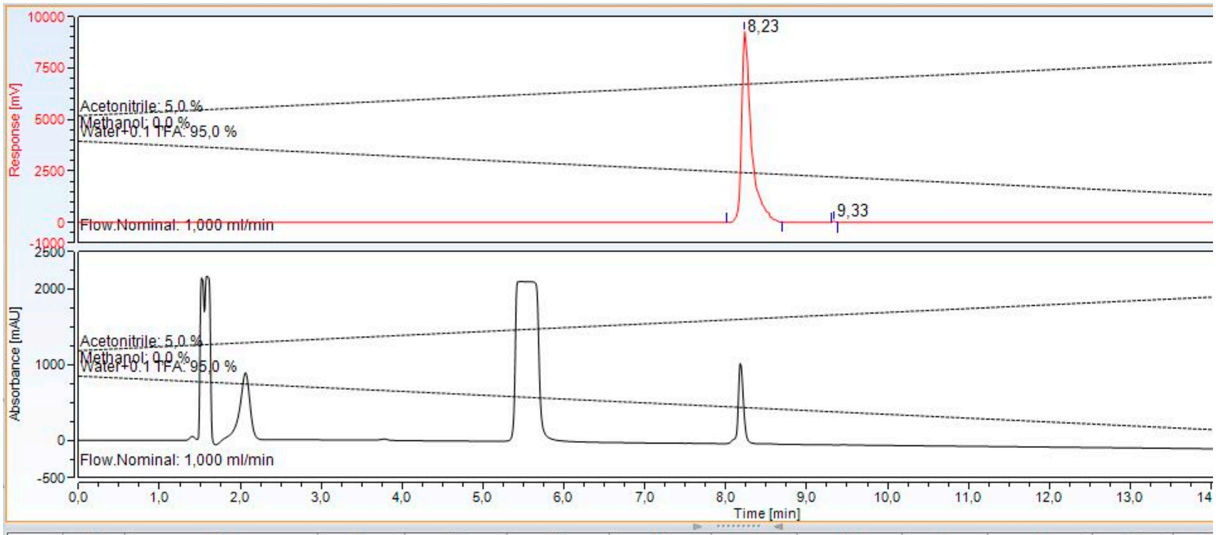

	A	B	C	D	E	F	G	H	I	J	K	L
1	Peak	Peak Name	Ret.Time	Rel.Area	Area	Height	Match	RSD Match	PPI	RSD PPI		
3	No.		min	%	mV*min	mV		%	nm	%		
4	1		8,233	99,82	1297,1562	9273,11	n.a.	n.a.	n.a.	n.a.		
5	2		9,333	0,18	2,3586	67,69	n.a.	n.a.	n.a.	n.a.		

Figure S24: Analytical HPLC of purified and formulated **[^{18}F]6A** co-injected with the non-radioactive reference **6A** (top: radioactive channel, bottom: UV channel 215 nm). Integration table refers to the radioactive channel. The broad and intense signal in UV is due to the sodium gentisate (80 mg/mL). Column Kinetex (phenomenex) H_2O/CH_3CN + 0.1% TFA 95/5 to 35/65 in 20 minutes at a flow rate of 1 mL/min.

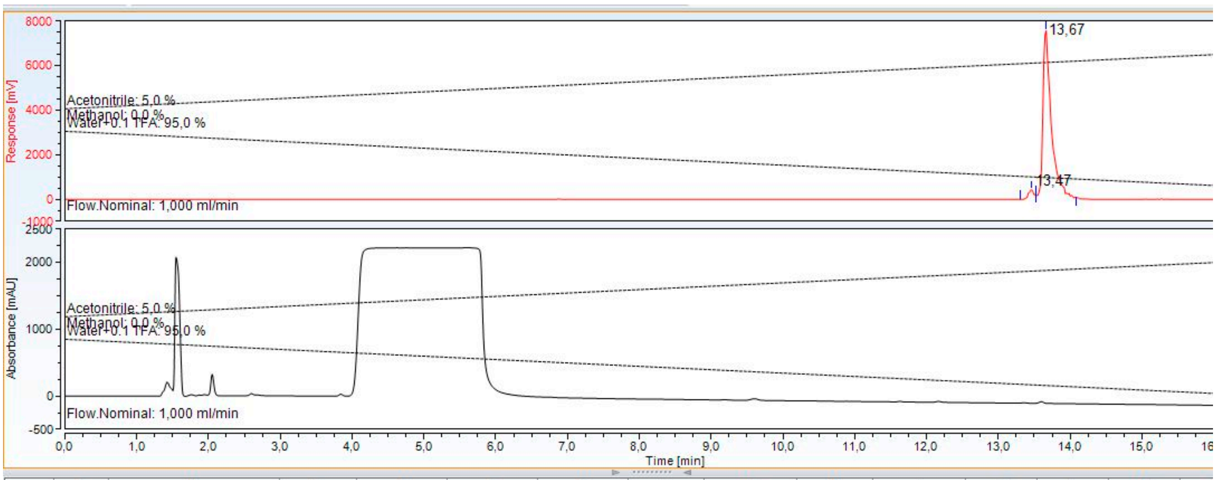

	A	B	C	D	E	F	G	H	I	J
1	Peak	Peak Name	Ret.Time	Rel.Area	Area	Height	Match	RSD Match	PPI	RSD PPI
3	No.		min	%	mV*min	mV		%	nm	%
4	1		13,467	1,33	12,9864	258,73	n.a.	n.a.	n.a.	n.a.
5	2		13,667	98,67	961,7537	7395,16	n.a.	n.a.	n.a.	n.a.

Figure S25: Analytical HPLC of purified and formulated **[18F]7A** (top: radioactive channel, bottom: UV channel 215 nm). Integration table refers to the radioactive channel. The broad and intense signal in UV is due to the sodium gentisate (80 mg/mL). Column Kinetex (phenomenex) H_2O/CH_3CN + 0.1% TFA 95/5 to 35/65 in 20 minutes at a flow rate of 1 mL/min.

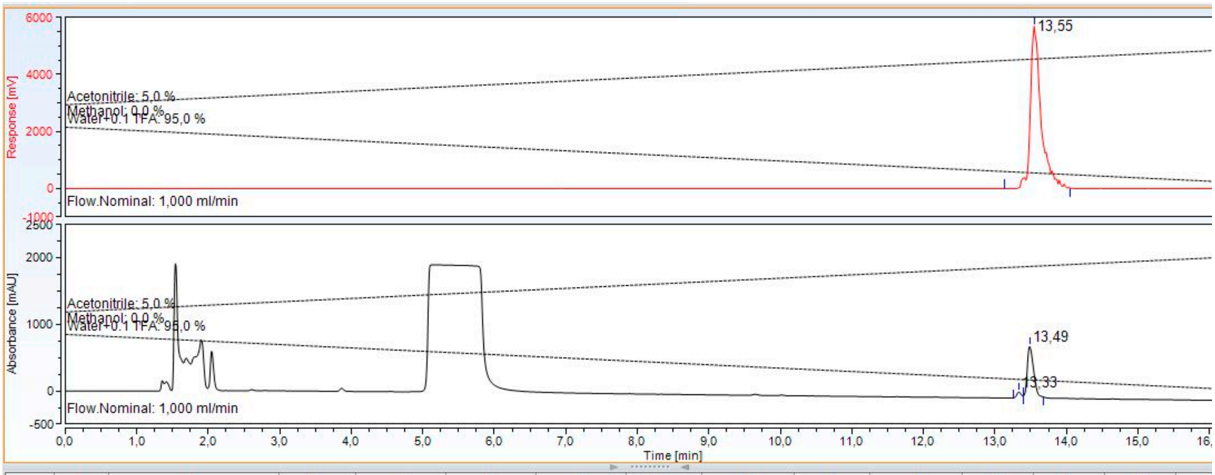

	A	B	C	D	E	F	G	H	I	J
1	Peak	Peak Name	Ret.Time	Rel.Area	Area	Height	Match	RSD Match	PPI	RSD PPI
3	No.		min	%	mV*min	mV		%	nm	%
4	1		13,550	100,00	980,5784	5681,75	n.a.	n.a.	n.a.	n.a.

Figure S26: Analytical HPLC of purified and formulated **[18F]7A** co-injected with the non-radioactive reference **6A** (top: radioactive channel, bottom: UV channel 215 nm). Integration table refers to the radioactive channel. The broad and intense signal in UV is due to the sodium gentisate (80 mg/mL). Column Kinetex (phenomenex) H_2O/CH_3CN + 0.1% TFA 95/5 to 35/65 in 20 minutes at a flow rate of 1 mL/min.

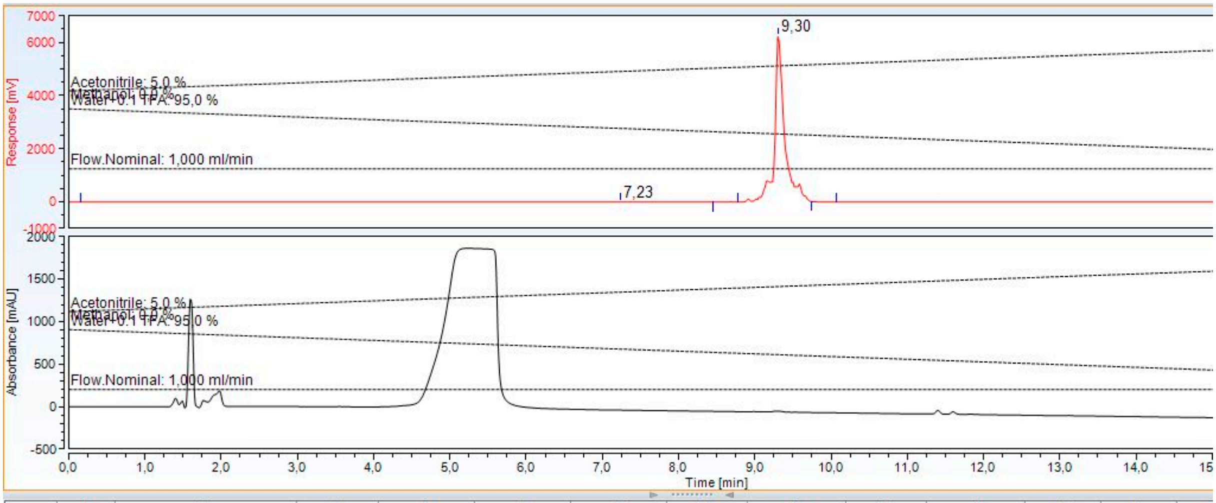

Peak No.	Peak Name	Ret.Time min	Rel.Area %	Area mV*min	Height mV	Match	RSD Match %	PPI nm	RSD PPI %
1		7,233	0,01	0,0773	0,02	999	0,09	217,8	0,16
2		9,300	99,95	950,4691	6208,06	951	2,33	222,3	1,50
3		21,700	0,04	0,3924	0,05	1000	0,01	215,0	0,03

Figure S27: Analytical HPLC of purified and formulated **[18F]8A** (top: radioactive channel, bottom: UV channel 215 nm). Integration table refers to the radioactive channel. The broad and intense signal in UV is due to the sodium gentisate (80 mg/mL). Column Kinetex (phenomenex) H_2O/CH_3CN + 0.1% TFA 95/5 to 35/65 in 20 minutes at a flow rate of 1 mL/min.

Stability of peptides [^{18}F]6A-8A in ready to inject solution 6 hours after the end of synthesis:

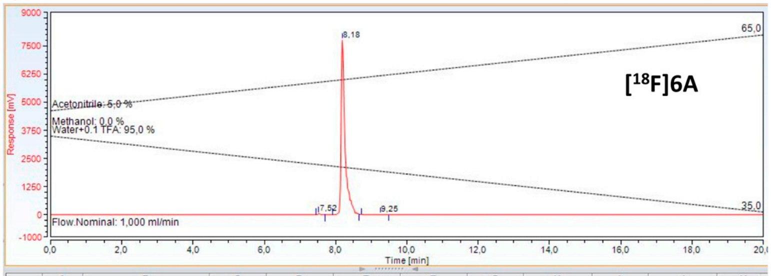

	A	B	C	D	E	F	G	H	I	J	K
1/3	Peak No.	Peak Name	Ret.Time min	Rel.Area %	Area mV*min	Height mV	Match	RSD Match %	PPI nm	RSD PPI %	
4	1		7,517	0,23	2,2599	48,76	n.a.	n.a.	n.a.	n.a.	
5	2		8,183	99,25	983,8082	7762,87	n.a.	n.a.	n.a.	n.a.	
6	3		9,250	0,52	5,1551	20,29	n.a.	n.a.	n.a.	n.a.	

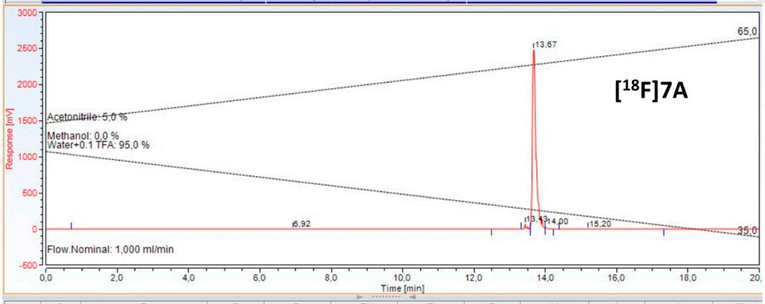

	A	B	C	D	E	F	G	H	I	J	K
1/3	Peak No.	Peak Name	Ret.Time min	Rel.Area %	Area mV*min	Height mV	Match	RSD Match %	PPI nm	RSD PPI %	
4	1		6,917	0,04	0,1258	2,90	n.a.	n.a.	n.a.	n.a.	
5	2		13,433	1,03	3,0102	67,73	n.a.	n.a.	n.a.	n.a.	
6	3		13,667	98,73	288,1997	2480,51	n.a.	n.a.	n.a.	n.a.	
7	4		14,000	0,16	0,4814	26,01	n.a.	n.a.	n.a.	n.a.	
8	5		15,200	0,03	0,1002	5,22	n.a.	n.a.	n.a.	n.a.	

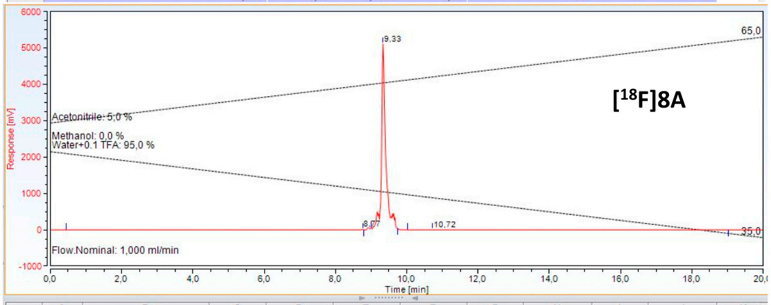

	A	B	C	D	E	F	G	H	I	J	K
1/3	Peak No.	Peak Name	Ret.Time min	Rel.Area %	Area mV*min	Height mV	Match	RSD Match %	PPI nm	RSD PPI %	
4	1		8,767	0,08	0,5234	14,72	727	41,35	295,9	8,06	
5	2		9,333	99,92	684,3108	5065,93	445	103,27	277,3	14,53	
6	3		10,717	0,01	0,0426	0,04	440	73,90	352,1	16,38	

Figure S28: Analytical HPLC of purified and formulated peptides (radioactive channel only) 6 hours after the end of synthesis. Top: **[^{18}F]6A**. Middle: **[^{18}F]7A**. Bottom: **[^{18}F]8A**. Column Kinetex (phenomenex) H_2O/CH_3CN + 0.1% TFA 95/5 to 35/65 in 20 minutes at a flow rate of 1 mL/min.

HPLC analyses of peptides [^{18}F]6B-8B (HDA)

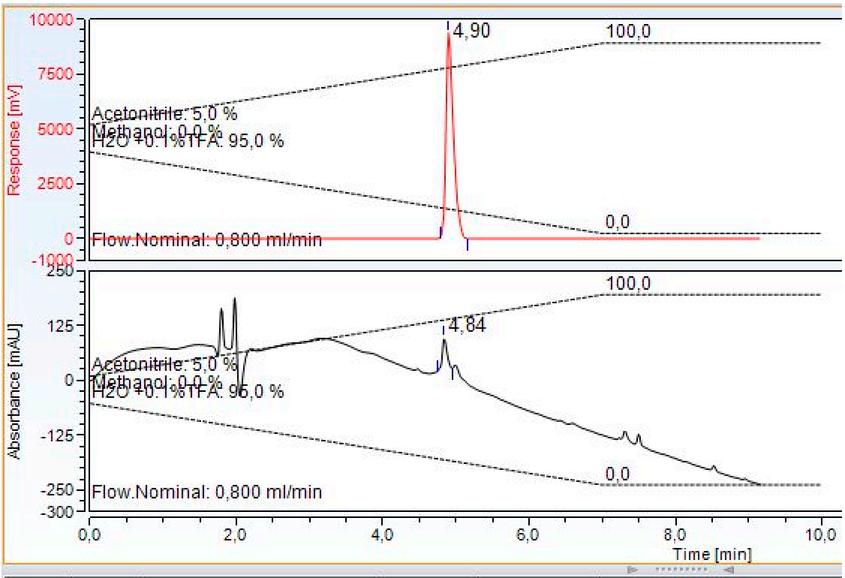

	A	B	C	D	E	F
1	Peak	Peak Name	Ret.Time	Rel.Area	Area	Height
3	No.		min	%	mV*min	mV
4	1		4,900	100,00	1101,0831	9365,78

Figure S29: Analytical HPLC of purified and formulated **[^{18}F]6B** (top: radioactive channel, bottom: UV channel 215 nm). Integration table refers to the radioactive channel. Column Kinetex (phenomenex) H_2O/CH_3CN + 0.1% TFA 95/5 to 0/100 in 7 minutes at a flow rate of 0.8 mL/min.

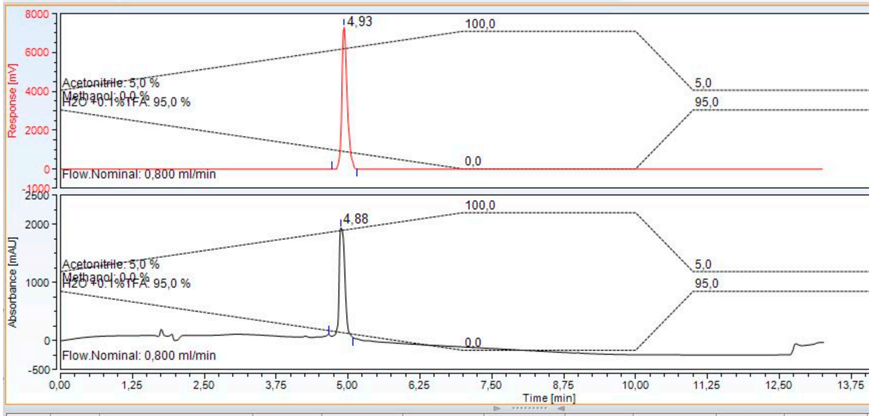

	A	B	C	D	E	F	G	H	I	J
1	Peak	Peak Name	Ret.Time	Rel.Area	Area	Height	Match	RSD Match	PPI	RSD PPI
3	No.		min	%	mV*min	mV		%	nm	%
4	1		4,933	100,00	784,1093	7296,06	973	2,40	222,6	1,50

Figure S30: Analytical HPLC of purified and formulated **[^{18}F]6B** co-injected with the non-radioactive reference **6B** (top: radioactive channel, bottom: UV channel 215 nm). Integration table refers to the radioactive channel. Column Kinetex (Phenomenex) H_2O/CH_3CN + 0.1% TFA 95/5 to 0/100 in 7 minutes at a flow rate of 0.8 mL/min.

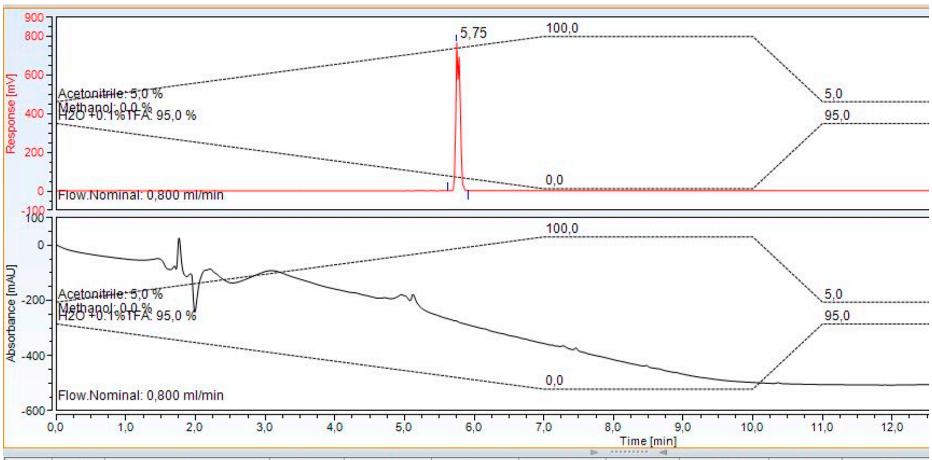

	A	B	C	D	E	F	G	H	I	J
1	Peak	Peak Name	Ret.Time	Rel.Area	Area	Height	Match	RSD Match	PPI	RSD PPI
3	No.		min	%	mV*min	mV		%	nm	%
4	1		5,750	100,00	52,6951	765,67	697	40,55	242,0	5,68

Figure S31: Analytical HPLC of purified and formulated **[^{18}F]7B** (top: radioactive channel, bottom: UV channel 215 nm). Integration table refers to the radioactive channel. Column Kinetex (Phenomenex) H_2O/CH_3CN + 0.1% TFA 95/5 to 0/100 in 7 minutes at a flow rate of 0.8 mL/min.

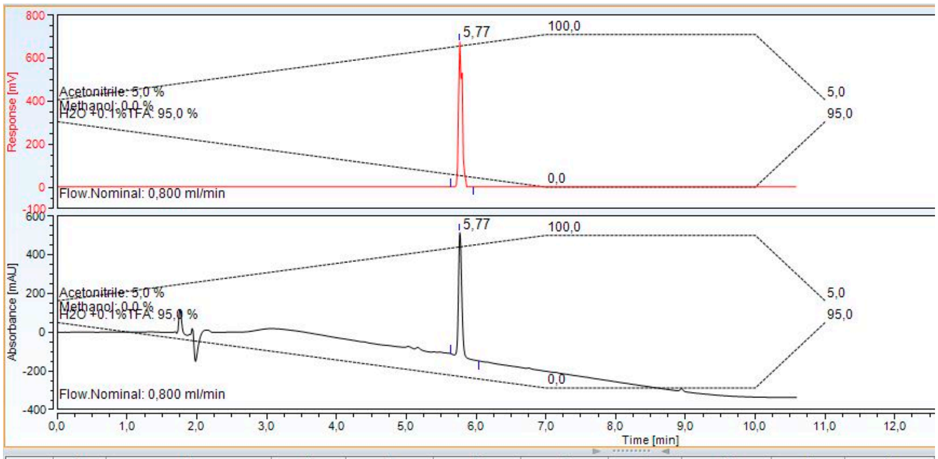

	A	B	C	D	E	F	G	H	I	J
1	Peak	Peak Name	Ret.Time	Rel.Area	Area	Height	Match	RSD Match	PPI	RSD PPI
3	No.		min	%	mV*min	mV		%	nm	%
4	1		5,767	100,00	41,1352	673,81	998	0,35	220,0	0,33

Figure S32: Analytical HPLC of purified and formulated **[^{18}F]7B** co-injected with the non-radioactive reference **7B** (top: radioactive channel, bottom: UV channel 215 nm). Integration table refers to the radioactive channel. Column Kinetex (Phenomenex) H_2O/CH_3CN + 0.1% TFA 95/5 to 0/100 in 7 minutes at a flow rate of 0.8 mL/min.

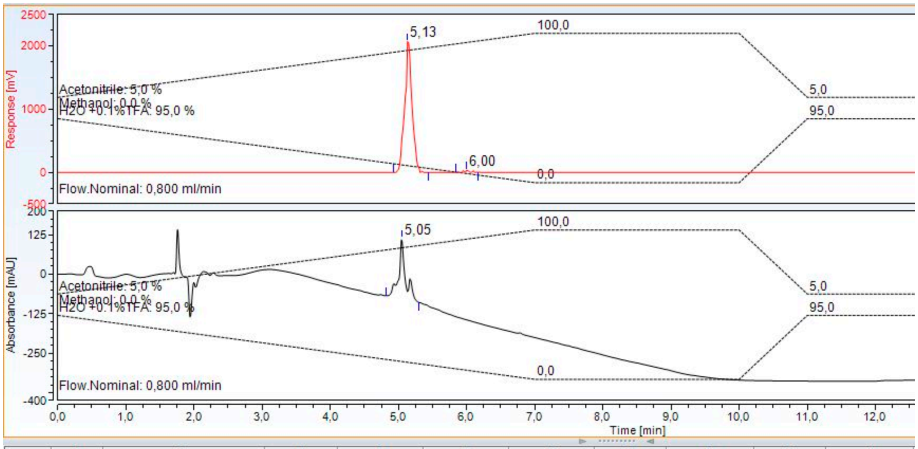

	A	B	C	D	E	F	G	H	I	J
1	Peak	Peak Name	Ret.Time	Rel.Area	Area	Height	Match	RSD Match	PPI	RSD PPI
3	No.		min	%	mV*min	mV		%	nm	%
4	1		5,133	99,00	259,7070	2068,36	335	66,78	235,2	13,12
5	2		6,000	1,00	2,6201	30,64	805	18,09	256,6	7,79

Figure S33: Analytical HPLC of purified and formulated **[^{18}F]8B** (top: radioactive channel, bottom: UV channel 215 nm). Integration table refers to the radioactive channel. Column Kinetex (phenomenex) H_2O/CH_3CN +0.1% TFA 95/5 to 0/100 in 7 minutes at a flow rate of 0.8 mL/min.

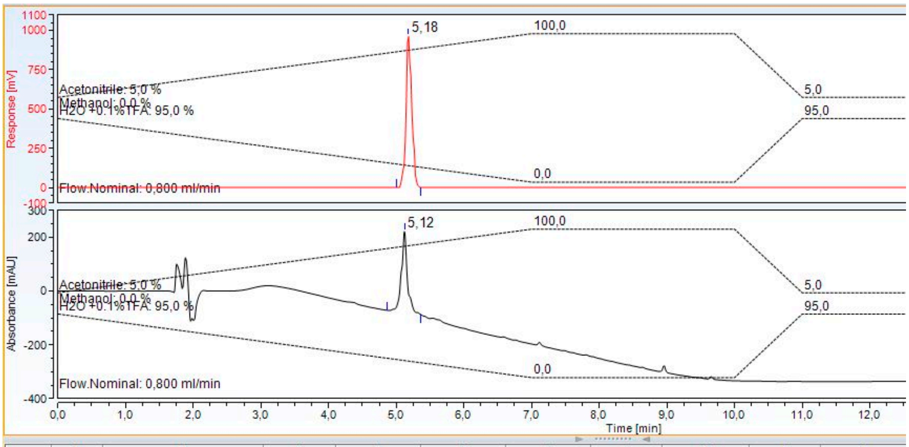

	A	B	C	D	E	F	G	H	I	J
1	Peak	Peak Name	Ret.Time	Rel.Area	Area	Height	Match	RSD Match	PPI	RSD PPI
3	No.		min	%	mV*min	mV		%	nm	%
4	1		5,183	100,00	95,4587	961,44	988	1,34	219,8	0,85

Figure S34: Analytical HPLC of purified and formulated **[^{18}F]8B** co-injected with the non-radioactive reference **8B** (top: radioactive channel, bottom: UV channel 215 nm). Integration table refers to the radioactive channel. Column Kinetex (Phenomenex) H_2O/CH_3CN + 0.1% TFA 95/5 to 0/100 in 7 minutes at a flow rate of 0.8 mL/min.

Stability of peptides [18F]6B-8B in ready to inject solution 6 hours after the end of synthesis:

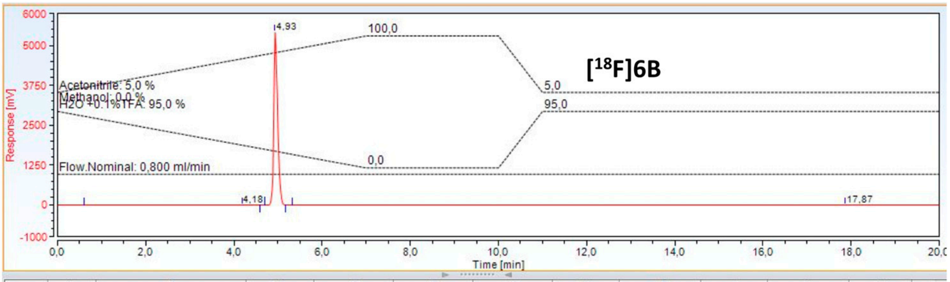

	A	B	C	D	E	F	G	H	I	J	K
1	Peak	Peak Name	Ret.Time	Rel.Area	Area	Height	Match	RSD Match	PPI	RSD PPI	
3	No.		min	%	mV*min	mV		%	nm	%	
4	1		4,183	0,00	0,0134	0,03	996	0,55	224,6	0,68	
5	2		4,933	99,98	549,4230	5411,77	989	1,88	219,4	0,39	
6	3		17,867	0,01	0,0736	0,05	994	0,66	367,5	4,05	

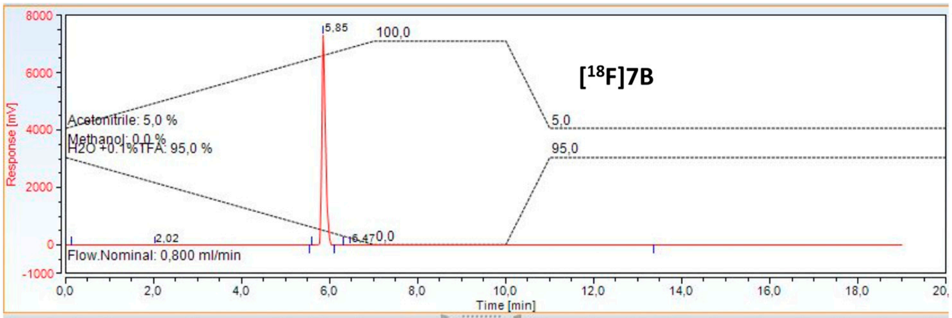

	A	B	C	D	E	F	G	H	I	J
1	Peak	Peak Name	Ret.Time	Rel.Area	Area	Height	Match	RSD Match	PPI	RSD P
3	No.		min	%	mV*min	mV		%	nm	%
4	1		2,017	0,01	0,0848	0,07	559	47,47	292,0	2
5	2		5,850	99,97	696,6359	7306,43	727	41,05	291,9	2
6	3		6,467	0,01	0,0976	0,01	1000	0,00	214,5	

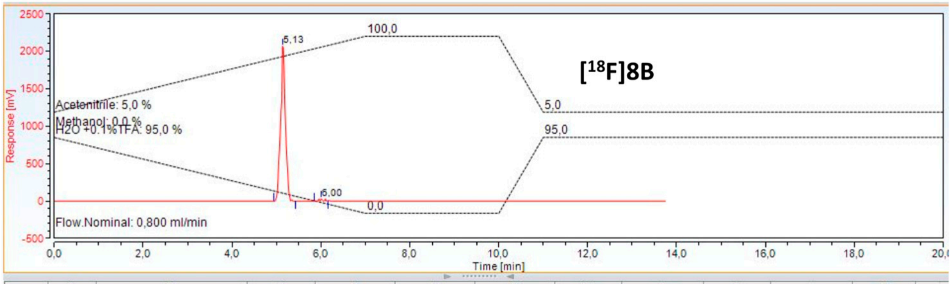

	A	B	C	D	E	F	G	H	I	J	K
1	Peak	Peak Name	Ret.Time	Rel.Area	Area	Height	Match	RSD Match	PPI	RSD PPI	
3	No.		min	%	mV*min	mV		%	nm	%	
4	1		5,133	99,00	259,7070	2068,36	335	66,78	235,2	13,12	
5	2		6,000	1,00	2,6201	30,64	805	18,09	256,6	7,79	

Figure S35: Analytical HPLC (Radioactive channel only) of purified and formulated peptides 6 hours after the end of synthesis. Top: **[18F]6B**. Middle: **[18F]7B**. Bottom: **[18F]8B**. Column Kinetex (Phenomenex) H_2O/CH_3CN + 0.1% TFA 95/5 to 0/100 in 7 minutes at a flow rate of 0.8 mL/min.
